# Rice OsPUB16 modulates the ‘SAPK9-OsMADS23-*OsAOC*’ pathway to reduce plant water-deficit tolerance by repressing ABA and JA biosynthesis

**DOI:** 10.1371/journal.pgen.1010520

**Published:** 2022-11-28

**Authors:** Qianlong Lv, Xingxing Li, Xinkai Jin, Ying Sun, Yuanyuan Wu, Wanmin Wang, Junli Huang

**Affiliations:** Key Laboratory of Biorheological Science and Technology, Ministry of Education, Bioengineering College, Chongqing University, Chongqing, China; Peking University, CHINA

## Abstract

Ubiquitin-mediated proteolysis plays crucial roles in plant responses to environmental stress. However, the mechanism by which E3 ubiquitin ligases modulate plant stress response still needs to be elucidated. In this study, we found that rice PLANT U-BOX PROTEIN 16 (OsPUB16), a U-box E3 ubiquitin ligase, negatively regulates rice drought response. Loss-of-function mutants of *OsPUB16* generated through CRISPR/Cas9 system exhibited the markedly enhanced water-deficit tolerance, while *OsPUB16* overexpression lines were hypersensitive to water deficit stress. Moreover, OsPUB16 negatively regulated ABA and JA response, and *ospub16* mutants produced more endogenous ABA and JA than wild type when exposed to water deficit. Mechanistic investigations revealed that OsPUB16 mediated the ubiquitination and degradation of OsMADS23, which is the substrate of OSMOTIC STRESS/ABA-ACTIVATED PROTEIN KINASE 9 (SAPK9) and increases rice drought tolerance by promoting ABA biosynthesis. Further, the ChIP-qPCR analysis and transient transactivation activity assays demonstrated that OsMADS23 activated the expression of JA-biosynthetic gene *OsAOC* by binding to its promoter. Interestingly, SAPK9-mediated phosphorylation on OsMADS23 reduced its ubiquitination level by interfering with the OsPUB16-OsMADS23 interaction, which thus enhanced OsMADS23 stability and promoted *OsAOC* expression. Collectively, our findings establish that OsPUB16 reduces plant water-deficit tolerance by modulating the ‘SAPK9-OsMADS23-*OsAOC*’ pathway to repress ABA and JA biosynthesis.

## Author summary

Plants face various adverse environmental stimuli during their growth, such as drought, salinity and extreme temperatures. Different from animals, plants cannot move, so they must endure abiotic stresses, which greatly limit the distribution of plants, alter their growth and development, and reduce crop productivity. To adapt to the major adverse environmental conditions, plants have developed multifaceted strategies at different levels. Among these, protein post-translational modifications play crucial roles. However, how protein post-translational modifications in response to environmental stress hormones modulate plant stress response still needs to be elucidated. Here, our results showed that the two types of post-translational modifications, phosphorylation and ubiquitination, regulate the balance between plant growth and stress response. Furthermore, there is the close link between the two types of post-translational modifications, which was affected by environmental stress hormones. The research will help us understand the mechanisms underlying the “trade-off” between plant growth and stress response, and engineer stress-resistant and high-yielding crops.

## Introduction

Drought stress inhibits plant growth and severely limits crop production [1]. Phytohormones abscisic acid (ABA) and jasmonic acid (JA) play important roles in promoting plant drought tolerance [2,3]. ABA is considered to be the main hormone that enhances plant drought tolerance through various morpho-physiological and molecular processes including stomata regulation, root development, and initiation of ABA-dependent pathway [4]. There is empirical evidence that the core ABA signaling components including PYRABACTIN RESISTANCE1 (PYR1)/PYR1-LIKE/REGULATORY COMPONENTS OF ABA RECEPTORS (PYR/PYL/RCARs), type 2C PROTEIN PHOSPHATASES (PP2Cs) and SNF1-RELATED KINASES 2 (SnRK2s) play pivotal roles in response to drought stress [5–7]. In the presence of ABA, ABA binding to PYR/PYL/RCAR receptors initiates deactivation of the co-receptor PP2Cs, thus resulting in the release of positive effector SnRK2s and subsequently activating downstream AREB/ABF transcription factors by mediating their phosphorylation [5,6,8]. During the process, the degradation of PP2Cs and PYR/PYL/RCARs mediated by E3 ubiquitin ligases finely modulates ABA signaling [9]. JA signaling regulates a broad range of JA-dependent responses including plant growth and stress response, and JASMONATE ZIM-DOMAIN (JAZ) proteins are primary transcriptional repressors in JA signaling pathway [10–12]. Like ABA and other phytohormone signaling pathways, JA signaling also follows a ‘Relief of Repression’ module that degrades the negative regulators via receptor-SCF-26*S* proteasome-mediated proteolysis. Briefly, JA is perceived by F-box protein CORONATINE INSENSITIVE1 (COI1), which recruits the E3 ubiquitin ligase to form complex SCF^COI1^ to target JAZ proteins, resulting in SCF^COI1^-dependent ubiquitination and degradation of JAZ proteins through the 26*S* proteasome [13,14]. The degradation of JAZ repressors alleviates the inhibition of downstream transcription factors, thus activating JA signaling [15,16].

A series of studies have shown that JA signaling can interact with ABA signaling to synergistically modulate multiple physiological processes including seed germination and biotic/abiotic stress response in plants [17–20]. In rice, OSMOTIC STRESS/ABA-ACTIVATED PROTEIN KINASEs (SAPKs), the SnRK2 homologues, are activated by ABA and hypertonic stress, and play essential roles in the transduction of ABA signaling [21,22]. Recent research has shown that ABA promotes JA biosynthesis through the ‘SAPK10-bZIP72-*OsAOC*’ pathway to synergistically inhibit rice seed germination [22]. In Arabidopsis, JAZ proteins modulate seed germination through the interaction with ABA-signaling transcription factors ABSCISIC ACID-INSENSITIVE 3 (ABI3) or ABI5 and thus repress their transactivation activity [20,23]. As a negative regulator of ABA signaling, GbWRKY1 reduces cotton salt and drought tolerance through an interaction network involving JAZ1 and ABI1 [24]. Although previous efforts have provided clues that ABA signaling coordination with JA signaling modulates plant seed germination and abiotic stress response, the molecular mechanism of the synergetic effect of ABA on JA signaling during plant drought response is still largely elusive.

The ubiquitin-proteasome system plays important roles in plant growth and adaptation to environmental stress [25]. Protein ubiquitination is a multistep reaction, requiring ubiquitin-activating enzyme E1, ubiquitin-conjugating enzyme E2 and ubiquitin ligase E3 [26]. Among these, the ubiquitin ligase E3 is critical in determining the specificity for degradation of target proteins [27]. The E3 ubiquitin ligases can be classified into HECT, RING, and U-box types based on the mechanisms for target recognition, ubiquitin tagging and specific domains [28–30]. Plant U-box (PUB) E3 ubiquitin ligases modulate various physiological processes and stress responses by regulating phytohormone signaling pathways [31,32]. In Arabidopsis, PUB12/13 modulate stomatal movement and confer drought response by mediating the degradation of ABI1, a key co-receptor of ABA [33], while they regulate cell differentiation and growth through a mechanism of membrane receptor kinase BRASSINOSTEROID-INSENSITIVE 1 (BRI1) internalization mediated by ubiquitination [34]. PUB18/PUB19 are negative regulators in ABA-mediated stomatal closure and drought stress responses [35]. PUB11 negatively modulates ABA-mediated drought response by the degradation of receptor-like protein kinases LEUCINE RICH REPEAT PROTEIN 1 (LRR1) and KINASE 7 (KIN7) [36]. In rice, OsPUB67 enhances drought tolerance by mediating a multilayered complex mechanism in an ABA-dependent manner [37]. Recently, PalPUB79 was indicated to enhance ABA-dependent drought tolerance via ubiquitination of PalWRKY77 in *Populus* [38]. Despite the recent advances, the knowledge about E3 ubiquitin ligases in monocot crops modulating abiotic stress response via phytohormone signalings is still limited.

Our previous study demonstrated that SAPK9-mediated phosphorylation on OsMADS23 conferred rice water-deficit tolerance in an ABA-dependent manner [39]. In this current study, we discovered that rice U-box E3 ubiquitin ligase OsPUB16 negatively regulates plant water-deficit tolerance via the ‘SAPK9-OsMADS23-*OsAOC*’ module by mediating ubiquitination and degradation of OsMADS23. OsMADS23 binds to the promoter region of JA-biosynthetic gene *OsAOC* to regulate JA biosynthesis, and SAPK9 enhances OsMADS23 protein stability as well as the transactivation activity by interfering with OsPUB16-OsMADS23 interaction via phosphorylation in an ABA-dependent manner. Thus, our findings provide a mechanistic understanding of how OsPUB16 modulates both ABA and JA signaling pathways that are integrated by the ‘SAPK9-OsMADS23-*OsAOC*’ module during the water-deficit response in rice.

## Results

### OsPUB16 is a U-box/ARM repeat protein localized in the cytoplasm and nucleus

**Fig 1:**
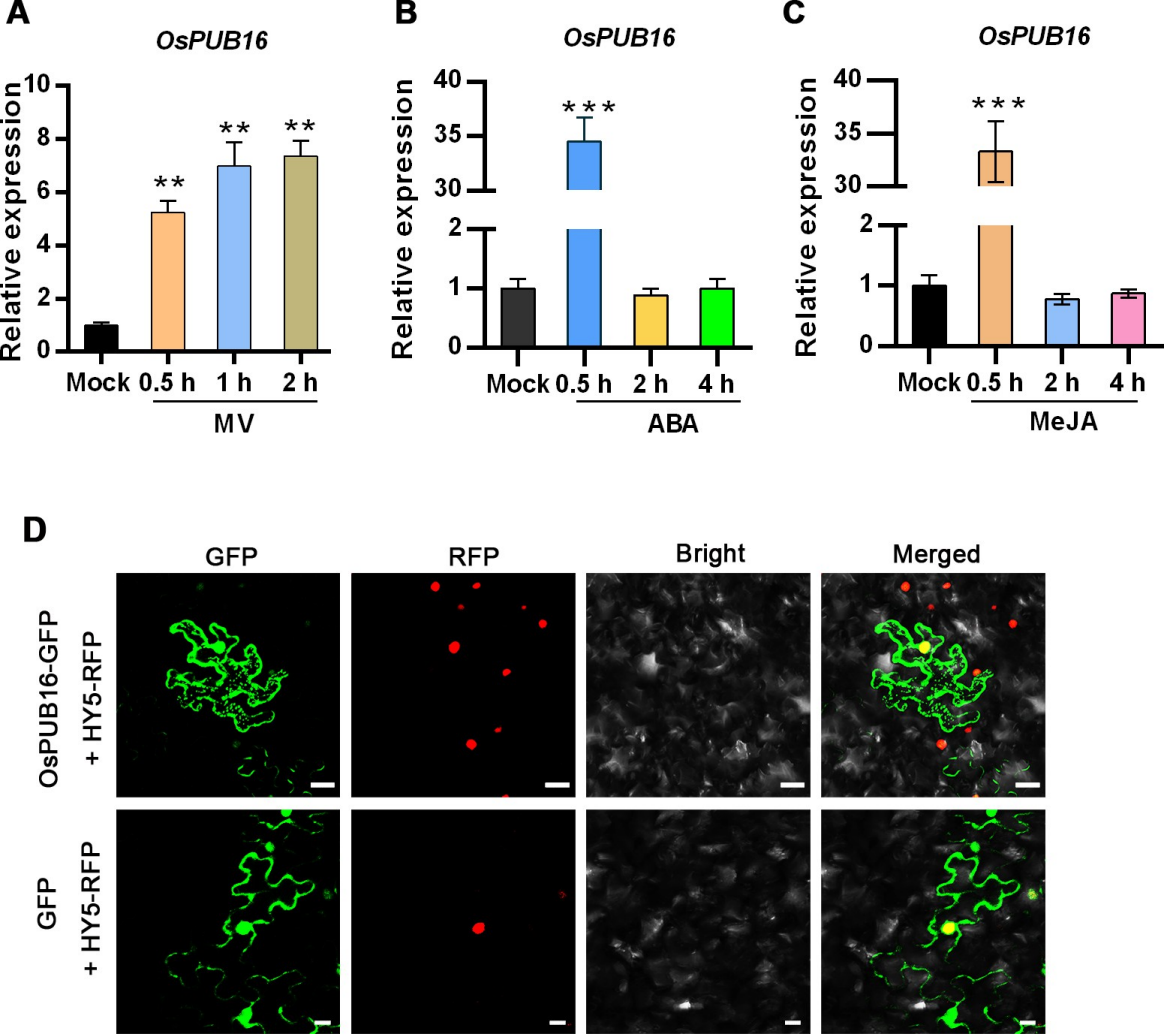
*OsPUB16* expression pattern and the subcellular localization of its protein. **(A-C)** *OsPUB16* expression in response to MV (methyl viologen) (A), ABA (B) and MeJA (C) at indicated time. Relative transcription levels of *OsPUB16* in the leaves of 2-week-old wild-type (Nipponbare) plants treated with 10 μM MV, 100 μM ABA or 100 μM MeJA for different time. Error bars indicate SD (*n* = 3 biological replicates). *OsActin1* was used as the internal control. The significant difference between mock and treatment was determined by Student’s *t* test. **p < 0.01, ***p < 0.001. **(D)** Subcellular localization of OsPUB16 in the epidermal cells of *Nicotiana benthamiana* leaves. GFP fluorescence signals were visualized using the confocal microscope (Leica SP8). HY5-RFP, the nuclear-localized marker. Scale bars, 40 μm.

To gain insight into the roles of rice U-box E3 ubiquitin ligases in plant growth and adaption to environmental stress, the phylogenetic analysis of a subset of U-box E3 proteins from different plants was performed. The result showed that the class-II U-box protein OsPUB16 shares high identity with OsPUB15 (S1 Fig), which regulates plant oxidative stress response and immunity in rice [40,41]. We further examined *OsPUB16* expression in response to MV (methyl viologen), an oxidative stress inducer in plants, as well as ABA and MeJA, which are two well-recognized environmental stress hormones. We found that the mRNA levels of *OsPUB16* were rapidly, strongly induced by MV and the two hormones (Fig 1A–1C). These results suggest that OsPUB16 is likely to play essential roles in plant response to oxidative stress caused by adverse environmental stimuli. In addition, analysis of the spatio-temporal expression profile using the PlaNet Browser [42] showed that *OsPUB16* was expressed in various tissues, but preferentially in the shoot apical meristem and young leaf (S2 Fig), implying that OsPUB16 may play important roles in plant growth. We investigated the subcellular localization of OsPUB16 by transiently expressing OsPUB16-GFP fusion protein in the epidermal cells of *Nicotiana benthamiana* leaves, and the result showed that OsPUB16 was mainly localized in the nucleus and cytoplasm (Fig 1D).

### OsPUB16 is required for plant growth in rice

The CRISPR/Cas9-mediated knockout mutants (*ospub16-1* and *ospub16-2*) were generated for investigating the biological functions of OsPUB16. The mutant *ospub16-1* harbored a one-nucleotide (T) insertion and *ospub16-2* harbored a 25-nucleotide deletion in the first exon of *OsPUB16*, respectively, which might result in frameshift mutations and thus early termination of protein translation (S3A and S3B Fig). Knockout of *OsPUB16* led to plant growth inhibition (S3C–S3F Fig). Notably, the homozygous *ospub16-2* mutant was lethal, probably due to earlier termination of protein translation caused by the large DNA fragment deletion in *ospub16-2* genome than that in *ospub16-1*, while the heterozygous *ospub16-2* mutant could survive with the reduced growth (S3C–S3F Fig). We then investigated *OsPUB16* expression in *ospub16* mutants, and the result showed that low levels of *OsPUB16* mRNA exist in *ospub16-2* (+/-) (S3G and S3H Fig), which is consistent with its reduced growth. Little mRNA of *OsPUB16* was detected in *ospub16-1* (S3G and S3H Fig). Together, these findings strongly demonstrated that *OsPUB16* is indispensable for plant growth and development in rice. We generated rice transgenic plants overexpressing *OsPUB16*. Contrary to *ospub16* mutants, the OsPUB16-OX plants (OX-4 and OX-10) exhibited the enhanced growth (S3I–S3K Fig).

### OsPUB16 acts as a negative regulator of plant water-deficit tolerance

**Fig 2:**
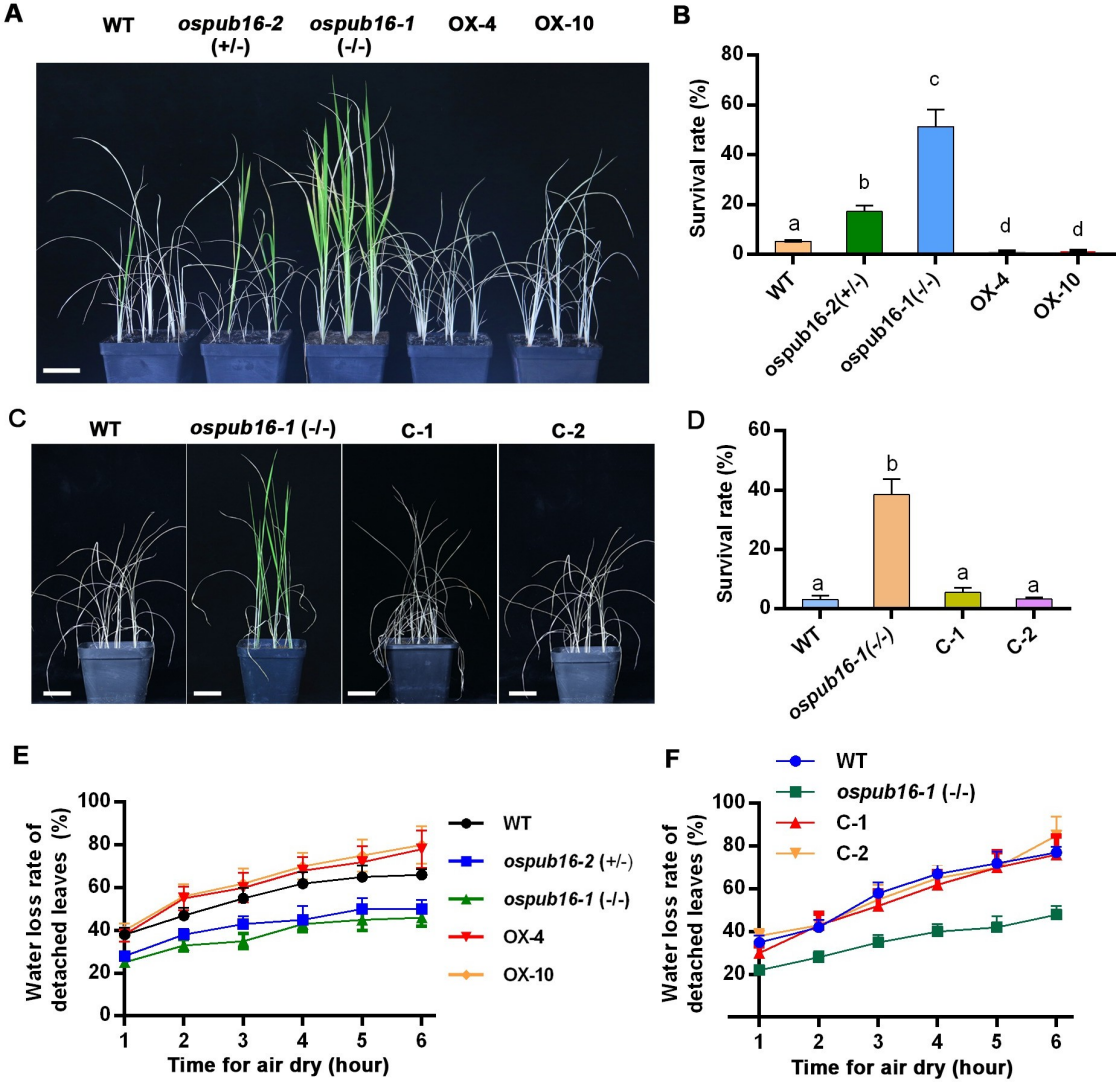
Response of the *ospub16* mutants and *OsPUB16* overexpression lines to water deficit stress. **(A)** Images showing the phenotypes of wild type (WT, Nipponbare), *ospub16* mutants and *OsPUB16* overexpression lines (OX-4, OX-10) after water deficit stress for 12 days and then rewatering for 5 days. Scale bars, 3 cm. **(B)** Survival rates of different genotypes after water deficit stress and then rewatering as described in (A). Data are means ± SD (*n*≥ 48). **(C, D)** Complementation test of *ospub16-1* mutant. WT, *ospub16-1* mutant and complementary lines (C-1, C-2) were exposed to deficit stress for 13 days and then rewatering for 4 days. Data are means ± SD (*n*≥ 27). Scale bars, 3 cm. **(E)** Water loss rates of detached leaves from 2-month-old plants. Data are means ± SD (*n* = 10). **(F)** Water loss rates of detached leaves from 1-month-old plants. Data are means ± SD (*n* = 10). In (B) and (D), two-way ANOVA followed by Bonferroni’s post-hoc test was performed. Different letters with the same superscript mark indicate significant differences (*p* < 0.05) between plants under the same conditions.

Next, the water-deficit stress response in wild type (WT), *ospub16* mutants and OsPUB16-OX plants was examined. Here, given that *ospub16-2* (-/-) mutants were lethal, the *ospub16-2* (+/-) mutants were used for the evaluation of water-deficit tolerance. After water withholding and rewatering, the survival rates of *ospub16*-*1* and *ospub16-2* (+/-) were 51.3% and 19.2%, respectively, which were significantly higher than that of WT (5.4%), whereas few OsPUB16-OX plants survived (Fig 2A and 2B). To further confirm the water-deficit tolerance in *ospub16* mutants was caused by *OsPUB16* knockout, we performed a functional complementation test by introducing the coding sequence of *OsPUB16* driven by its own promoter into the *ospub16-1* mutant, and the positive transgenic lines harboring the *OsPUB16*_*pro*_:*OsPUB16* (C-1, C-2) showed WT phenotypes, indicated by the reduced water deficit tolerance (Figs 2C, 2D and S4). Further, the water loss rates of detached leaves showed that *ospub16* mutants lost water more slowly but overexpression lines lost water faster than WT (Fig 2E), which supported the result that *ospub16* mutants were more tolerant but overexpression lines were more sensitive to water deficit stress. In agreement, the complementary lines of *ospub16-1* restored the rapid water loss (Fig 2F). The findings suggest that OsPUB16 negatively regulates the water-deficit tolerance in rice.

**Fig 3:**
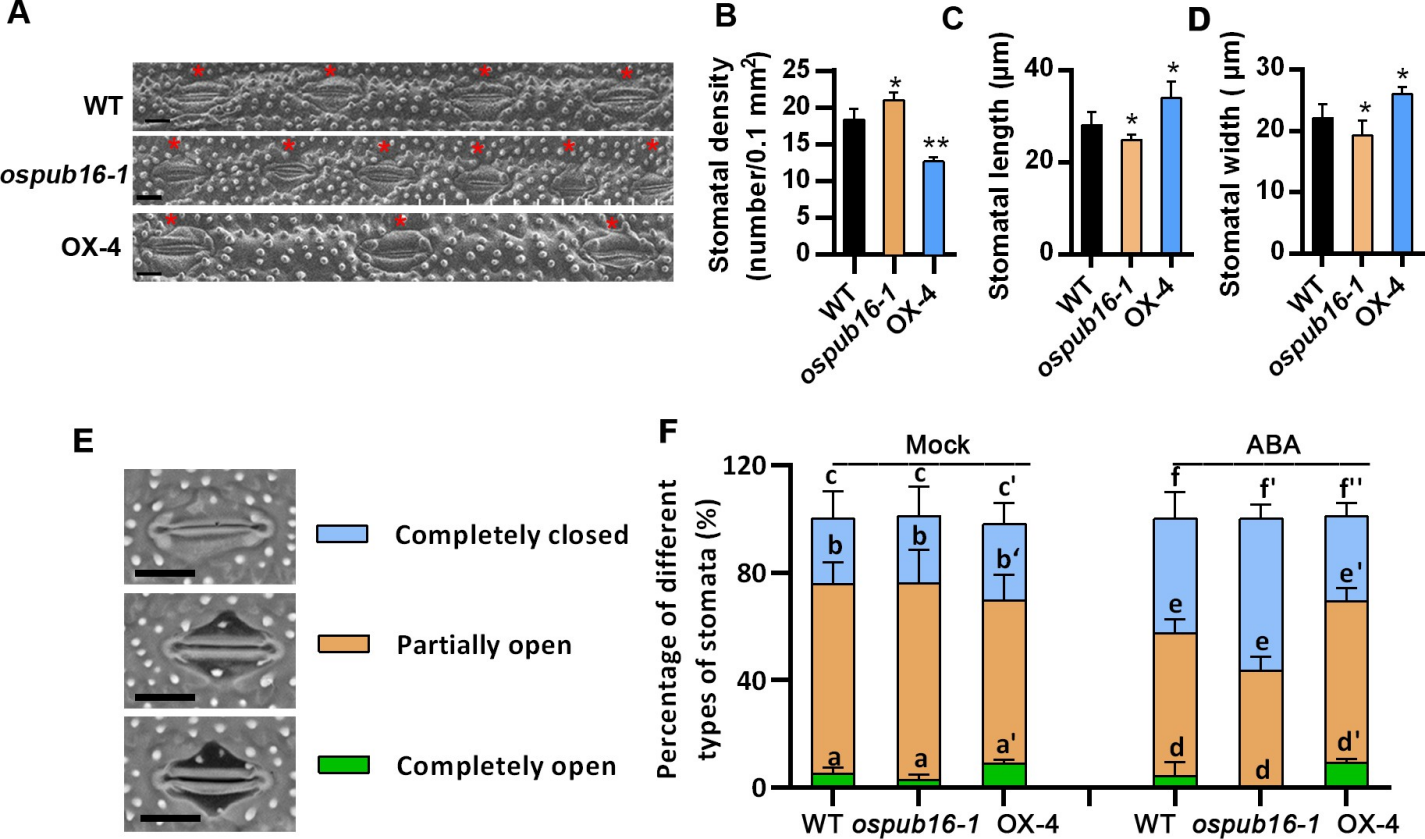
Effects of OsPUB16 on the leaf stomatal development and response to ABA. **(A)** Stomata of different genotypes on leaves. Scale bars, 50 μm. The stomata are marked with red asterisks. **(B-D)** Comparisons of stomatal density, length and width between WT and the transgenic lines, respectively. Data are means ± SD (*n*≥ 200, from 5 independent plants). (**E**) Images showing the completely open, partially open, and completely closed stomata in rice leaves. **(F)** Percentages of completely open, partially open, and completely closed stomata in different genotypes with or without ABA treatment. Data are means ± SD (*n*≥ 200, from 5 independent plants). In (B-D), the significant difference between transgenic lines and WT was determined by Student’s *t* test. *p < 0.05, **p < 0.01 or ***p < 0.001. In (F), two-way ANOVA followed by Bonferroni’s post-hoc test was performed. Different letters with the same superscript mark indicate significant differences (*p* < 0.05) between plants under the same conditions.

Stomatal density, size and morphology in leaf are closely associated with the leaf water loss rate under water deficit conditions [43]. To explore the possible cellular processes regulated by OsPUB16 during plant water-deficit response, we then examined the influence of *OsPUB16* knockout or overexpression on the stomatal development in rice leaves. Interestingly, we found that the stomatal density was increased in *ospub16-1*, but reduced in OX-4 leaves, in comparison with that in WT (Fig 3A and 3B). However, a significant decrease in stomatal length and width was observed in *ospub16-1* mutant (Fig 3C and 3D), which supports that *ospub16-1* lost water more slowly when water was withheld (Fig 2E). By contrast, overexpression of *OsPUB16* led to the increased stomatal size (Fig 3C and 3D), suggesting a rapid water loss in OsPUB16-OX plants under water deficit conditions. ABA-induced stomatal closure is a well-recognized model system for the study of plant response to drought stress [44]. We then compared the ABA-induced stomatal movement in WT, *ospub16* mutant and OsPUB16-OX leaves. Under daylight conditions, stomata in rice leaf can be classified into three typical status categories: completely open, partially open, and completely closed (Fig 3E). Without ABA, there was little difference in the proportions of these three categories of stomata between WT and *ospub16-1* plants (Fig 3F). However, in the presence of ABA, more stomata were completely closed and fewer stomata were completely open in *ospub16-1* leaves than that in WT. Expectedly, stomatal movement exhibited the opposite trend in OsPUB16-OX leaves (Fig 3F). Overall, these results demonstrate that OsPUB16 negatively modulates plant water-deficit tolerance, possibly through regulating stomatal development and ABA-induced stomatal movement.

### OsPUB16 attenuates the ABA and JA response by repressing their biosynthesis

**Fig 4:**
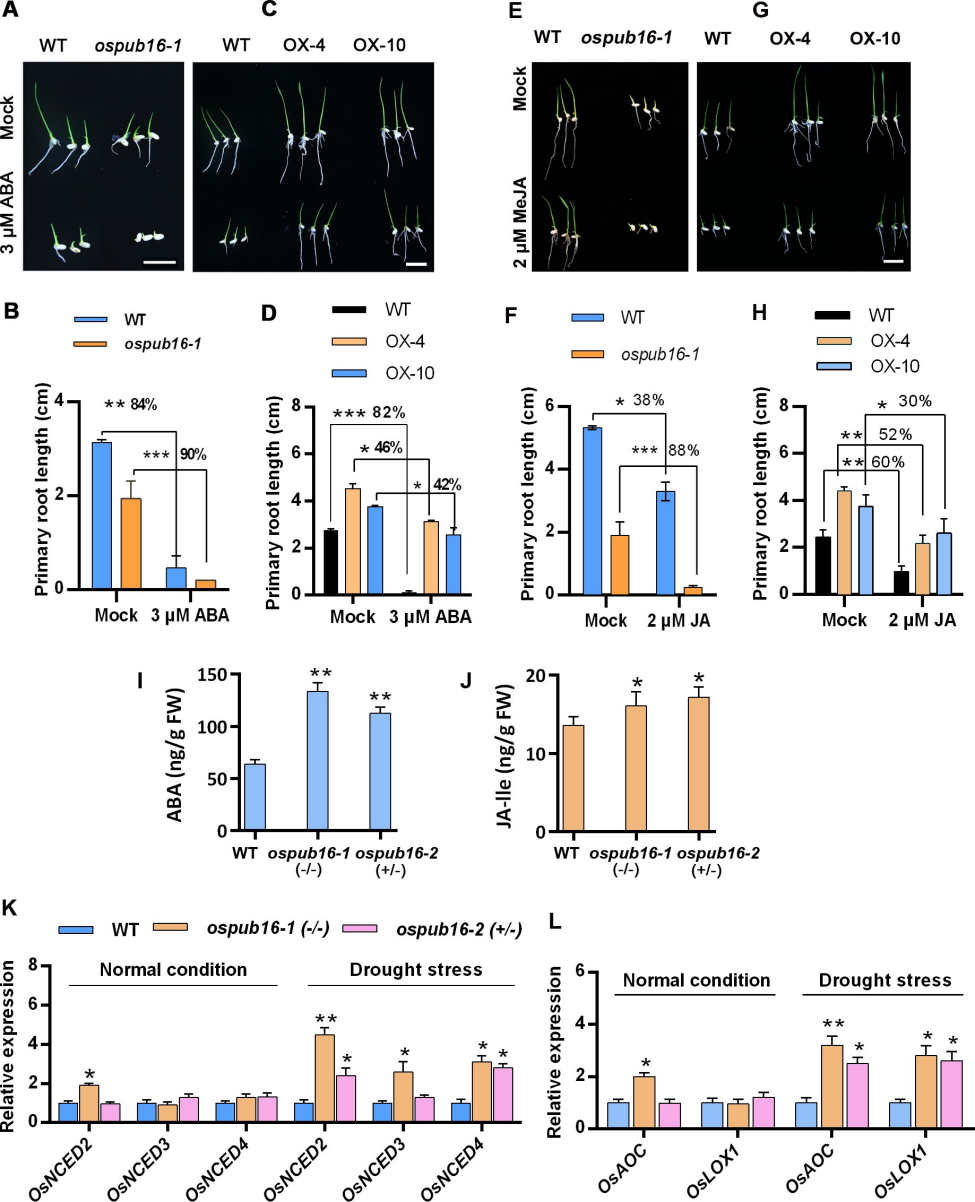
OsPUB16 reduced the ABA and JA response in rice. **(A, C)** Images showing the responses of *ospub16-1* mutant, wild type (WT, Nipponbare) and *OsPUB16* overexpression lines (OX-4 and OX-10) to exogenous ABA. Scale bars, 2 cm. Uniformly germinated seeds were grown on 1/2MS medium supplemented with or without 3 μM ABA for 3 days. **(B, D)** Measurement of the primary root length of plants as described in (A) and (C), respectively. Data are means ± SD (*n* = 20). **(E, G)** Images showing the responses of different genotypes to exogenous MeJA. Scale bars, 2 cm. Uniformly germinated seeds were grown on 1/2MS medium with or without 2 μM MeJA for 3 days. **(F, H)** Measurement of the primary root length of plants as described in (E) and (G), respectively. Data are means ± SD (*n* = 15). **(I, J)** ABA and JA-lle content in WT and *ospub16* mutants after exposed to water deficit stress. Two-week-old plants were subjected to water deficit stress for 5 days, and leaves were collected for measurement of bioactive ABA and JA-lle content. Data are means ± SD (*n* = 3). **(K, L)** Expression of key genes involved in ABA (K) and JA (L) biosynthesis in *ospub16* mutants and WT under normal conditions and drought stress. Ten-day-old plants were subjected to water deficit stress for 3 days, and leaf examples were collected. Data are means ± SD (*n* = 3 biological replicates). *OsActin1* was used as the internal control. The transcript levels in WT were defined as “1”. The significant difference was determined by Student’s *t* test. *p < 0.05, **p < 0.01 or ***p < 0.001.

ABA and JA are considered as the main hormones that promote drought tolerance in plants through regulating various physiological responses and molecular processes [4,44,45]. Given the high expression of *OsPUB16* induced by ABA and MeJA (Fig 1B and 1C), we asked whether OsPUB16 regulates ABA and JA responses in plants. We first investigated the response of different genotypes to exogenous ABA and MeJA, respectively, through the primary root growth inhibition assay. As shown in Fig 4A–4D, the primary root elongation in *ospub16-1* mutant was inhibited more severely by ABA treatment, indicated by the higher decrease rate in root length than that in WT; however, the inhibitory effects of ABA on the root growth of OsPUB16-OX plants were poorer than that on WT. Similar response was observed when plants were exposed to exogenous MeJA (Fig 4E–4H). The ABA and JA sensitivity of *ospub16* mutants was further confirmed by the seed germination inhibition assay (S5 Fig). Thus, these results demonstrated that *ospub16* mutants were hypersensitive, while OsPUB16-OX plants were hyposensitive to ABA and JA, suggesting that OsPUB16 inhibits the ABA and JA response. Further, the endogenous ABA and JA levels in WT and *ospub16* plants exposed to water deficit stress were evaluated. As shown in Fig 4I and 4J, after water withholding for 5 days, both ABA and JA-lle levels were much higher in *ospub16* plants than that in WT. Expectedly, the expression of ABA and JA biosynthetic genes in *ospub16* mutants was significantly increased when exposed to drought stress (Fig 4K and 4L). These results imply that OsPUB16 represses plant ABA and JA response possibly through inhibiting the biosynthesis of two phytohormones.

### OsPUB16 physically interacts with a subset of JAZ proteins, and mediates the OsJAZ9 protein ubiquitination and degradation

**Fig 5:**
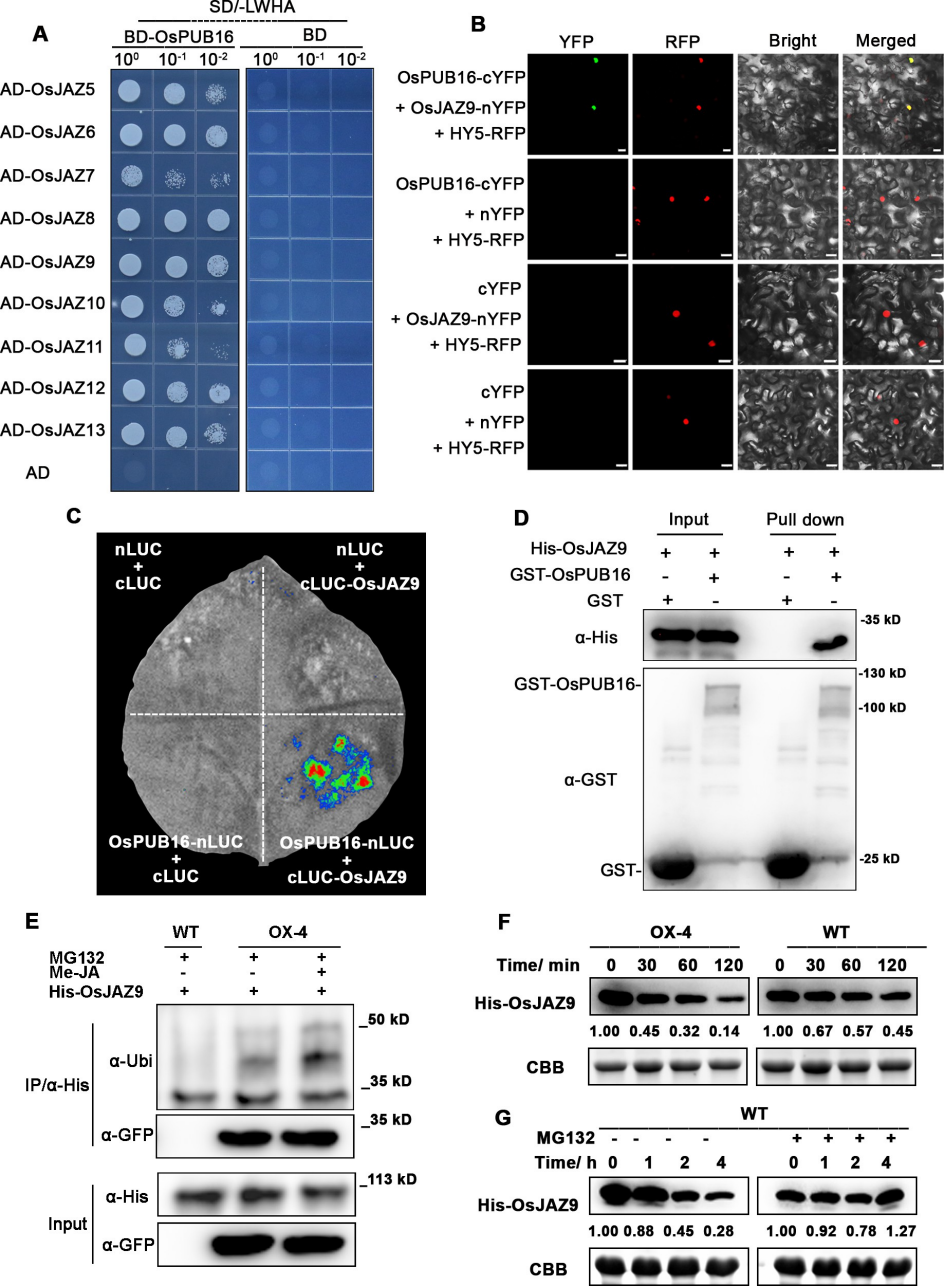
OsPUB16 interacts with rice JAZ proteins, and mediates ubiquitination and degradation of OsJAZ9. **(A)** Yeast two-hybrid assays to test the interaction of OsPUB16 with rice JAZ proteins. Yeast cells were grown on synthetic defined (SD)/-Ade-His-Leu-Trp (-LWHA) medium for 2 days. **(B)** Bimolecular fluorescence complementation (BiFC) assay for the interaction of OsPUB16 with OsJAZ9 in *Nicotiana benthamiana* epidermal cells. YFP, yellow fluorescent protein. RFP, red fluorescent protein. HY5-RFP, the nuclear-localized marker. Scale bars, 20 μm. **(C)** OsPUB16 interacts with OsJAZ9 assessed by firefly luciferase complementation imaging (LCI) assays in *N*. *benthamiana* leaves. **(D)** OsPUB16 interacts with OsJAZ9 indicated by the GST Pull-down assay. GST-OsPUB16 was used as bait, and pull-down of His-OsJAZ9 was detected by anti-His antibody. GST-OsPUB16 and GST were detected by anti-GST antibody. **(E)** Semi-endogenous ubiquitination analysis showing the ubiquitination of OsJAZ9 mediated by OsPUB16, with or without MeJA. Briefly, the total protein extracts from wild type (WT) or *OsPUB16*-overexpressing plants (OX-4) were incubated with His-OsJAZ9 with or without 100 μM MeJA, and then the immunoprecipitation was performed with anti-His antibodies. The immunoprecipitated proteins were detected with different antibodies. Three independent experiments were performed, and representative pictures were shown. **(F)** Cell-free degradation assays for recombinant His-OsJAZ9 protein in the protein extracts from WT or *OsPUB16*-overexpressing plants (OX-4), in the presence of ATP. The numbers at indicated times show the relative levels of the recombinant protein. Three independent replicate experiments were performed, and representative pictures were given. The Coomassie blue-stained ribulose-1,5-bisphosphate carboxylase/oxygenase (Rubisco) large subunit (Rbc L) was used as a loading control. **(G)** Cell-free degradation assays for recombinant His-OsJAZ9 in the protein extracts from WT with or without protease inhibitor MG132, in the presence of ATP. The numbers at indicated times indicate the relative levels of the recombinant protein from three independent replicate experiments. In cell-free degradation assays, equal amount of His-OsJAZ9 protein was incubated with equal amount of plant protein extracts, in the presence of 10 mM ATP. His-OsJAZ9 was detected by immunoblot analysis with anti-His antibody. Three independent replicate experiments were performed, and representative pictures were given.

JA signaling is associated with plant drought stress response, and the JAZ proteins act as key repressors of JA signaling to block the downstream responses through the interaction with the transcription factors [46]. To further explore the roles of OsPUB16 in JA signaling, we checked whether OsPUB16 interacts with JAZ proteins through the yeast two-hybrid (Y2H) assay. Interestingly, we found that OsPUB16 interacted with multiple members of the JAZ family proteins (Fig 5A), suggesting that OsPUB16 is involved in JA signaling. Here we selected OsJAZ9 as the representative of JAZ family members for further validation for the interaction of OsPUB16 with JAZ proteins. In the bimolecular fluorescence complementation (BiFC) assay, strong YFP fluorescence signal was observed in the nucleus of epidermal cells of *N*. *benthamiana* leaves co-expressing OsPUB16-cYFP and OsJAZ9-nYFP, but no signal was detected when each construct was co-expressed with an empty vector (Fig 5B). Next, we used the firefly luciferase complementation imaging (LCI) assay to confirm the interaction. Consistently, no luciferase signal was detected in *N*. *benthamiana* leaves harboring each construct and an empty vector. However, we detected a robust luciferase signal in the presence of OsPUB16-nLUC and cLUC-OsJAZ9 (Fig 5C). The GST pull-down assay also demonstrated that GST-OsPUB16, but not GST alone, pulled down a significant amount of His-OsJAZ9 (Fig 5D). Based on these observations, we conclude that OsPUB16 interacts with OsJAZ9 in *vitro* and *in vivo*.

It is recognized that E3 ubiquitin ligases mediate the ubiquitination and subsequent degradation of substrate proteins by 26*S* proteasome system. The OsPUB16-OsJAZ9 interaction suggests that OsJAZ9 might be a substrate of OsPUB16. Next, we investigated whether OsPUB16 mediates the ubiquitination on OsJAZ9, and how JA affects the ubiquitination process. Recombinant His-OsJAZ9 was incubated with protein extracts from WT or OsPUB16-OX plants, with or without 100 μM MeJA, and then immunoprecipitation was performed with anti-His beads. Detection of the immunoprecipitated His-OsJAZ9 by anti-Ubi antibody showed that, compared to protein extracts from WT plants, the extracts from OsPUB16-OX plants increased the ubiquitination on His-OsJAZ9, and the ubiquitination levels were further enhanced in the presence of MeJA (Fig 5E). These findings demonstrated that OsPUB16 mediated the ubiquitination on OsJAZ9 in a JA-dependent manner. We then performed the cell-free degradation assay by using His-OsJAZ9 protein. His-OsJAZ9 protein was incubated with equal amount of total protein extracts from WT or OsPUB16-OX plants, in the presence of ATP. Immunoblot analysis showed that His-OsJAZ9 was degraded much faster in OsPUB16-OX extracts than that in WT extracts (Fig 5F), indicating that OsPUB16 mediates His-OsJAZ9 degradation. However, there was no significant difference in the degradation rate for His-OsJAZ9 in *ospub16-1* extracts, compared to that in WT (S6 Fig), probably due to the compensational effects by other U-box E3 ubiquitin ligases in *ospub16-1* mutant. His-OsJAZ9 degradation in WT extracts was largely blocked by an addition of MG132, a 26*S* proteasome inhibitor (Fig 5G), indicating that OsPUB16 mediates His-OsJAZ9 degradation mainly through the 26*S* proteasome system. These results indicate that OsPUB16 can regulate JA signaling by mediating JAZ protein degradation.

### OsPUB16 physically interacts with OsMADS23, leading to its ubiquitination and degradation

**Fig 6:**
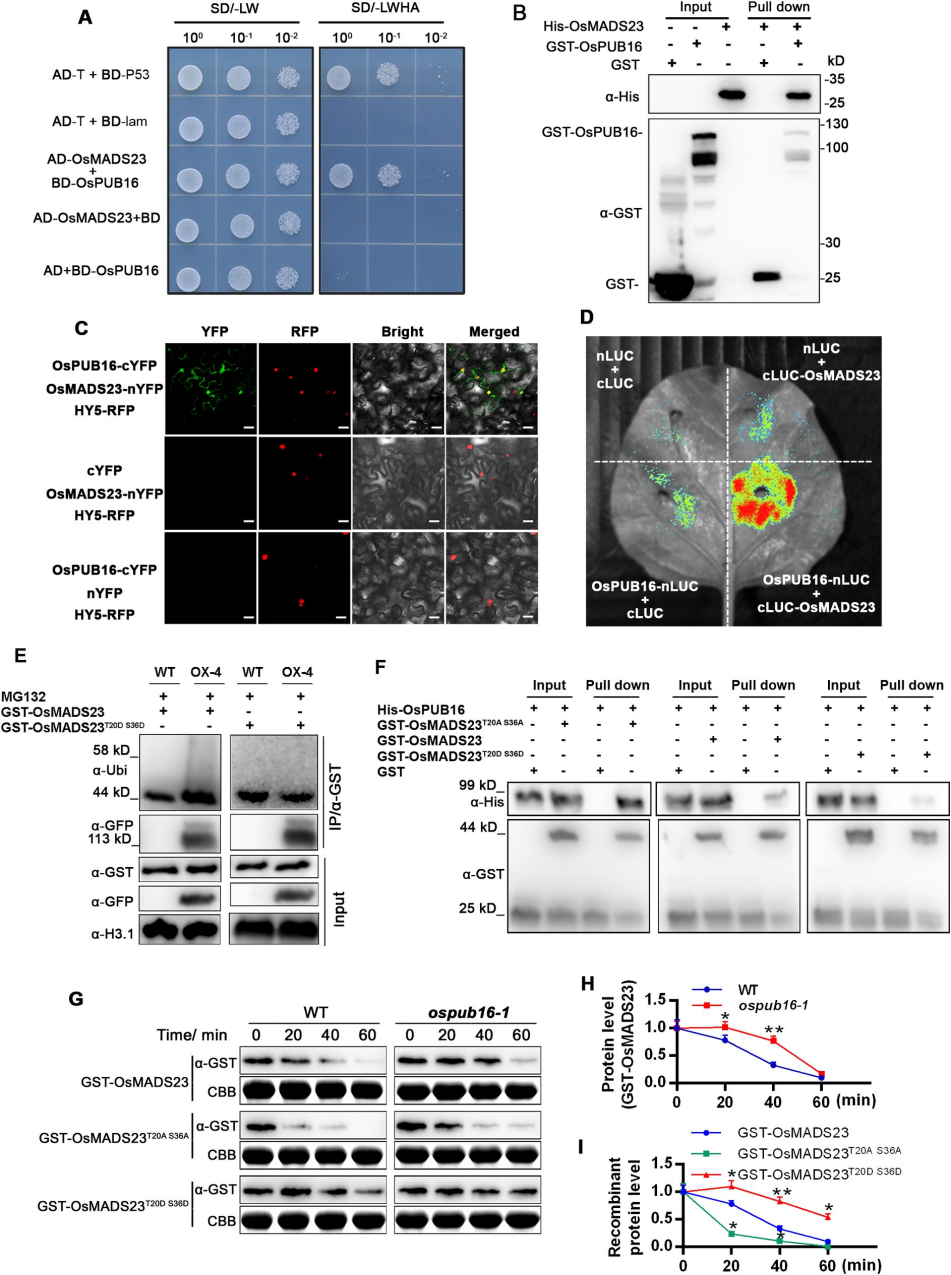
OsPUB16 mediates the ubiquitination and degradation of OsMADS23 via the physical interaction. **(A)** Yeast two-hybrid assays showing the interaction between OsPUB16 and OsMADS23. Yeast cells were grown on synthetic defined (SD)/-Leu-Trp (-LW) medium and SD/-Ade-His-Leu-Trp (-LWHA) medium. The AD-T + BD-P53 was used as a positive control, and AD-T + BD-lam as a negative control. **(B)** GST Pull-down assay showing the interaction of OsPUB16 with OsMADS23. GST-OsPUB16 was used as bait, and pulled-down His-OsMADS23 was detected by anti-His antibody. GST-OsPUB16 and GST were detected by anti-GST antibody. **(C)** Bimolecular fluorescence complementation (BiFC) assay for the interaction of OsPUB16 with OsMADS23 in *Nicotiana benthamiana* epidermal cells. YFP, yellow fluorescent protein. RFP, red fluorescent protein. HY5-RFP, the nuclear-localized marker. Scale bars, 20 μm. **(D)** Firefly luciferase complementation imaging (LCI) assay showing the interaction of OsPUB16 with OsMADS23 in *N*. *benthamiana* leaves. **(E)** Semi-endogenous ubiquitination analysis showing that phosphorylation of OsMADS23 blocks its ubiquitination mediated by OsPUB16. Briefly, the total protein extracts from wild type (WT) or *OsPUB16*-overexpressing plants (OX-4) plants were incubated with GST-OsMADS23 or GST-OsMADS23^T20D S36D^ for 5 hours, in the presence of 50 μM MG132, and then the immunoprecipitation was performed with anti-GST beads. The immunoprecipitated proteins were detected with anti-Ubi and anti-GFP antibodies, respectively. The immunoblot analysis of Histone H3.1 (H3.1) was used as a loading control of protein extracts. Three independent experiments were performed, and representative pictures were shown. **(F)** GST Pull-down assays showing the interaction of GST-OsMADS23 or its different mutated versions (GST-OsMADS23^T20A S36A^ and GST-OsMADS23^T20D S36D^) with His-OsPUB16. **(G-I)** OsPUB16 promotes the degradation of OsMADS23 in the cell-free degradation assay. Recombinant GST-OsMADS23 or its different mutated versions was incubated in equal amount of total protein extracts from two-week-old wild type (WT) and *ospub16*-1 mutant in the presence of 10 mM ATP. GST-OsMADS23 and its mutated versions were detected by immunoblot analysis using anti-GST antibody. The Coomassie blue–stained ribulose-1,5-bisphosphate carboxylase/oxygenase (Rubisco) large subunit (Rbc L) was used as a loading control. Representative pictures were shown in (G) and relative protein levels were analyzed in (H, I). In (H) and (I), data are means ± SD (*n* = 3, three independent replicate experiments). The significant difference between WT and *ospub16-1* mutant, or native GST-OsMADS23 and its different mutated versions was determined by Student’s *t* test. *p < 0.05, **p < 0.01 or ***p < 0.001.

OsPUB16 functions as a negative regulator in the water-deficit tolerance (Fig 2), and our recent study revealed that SAPK9-mediated phosphorylation on OsMADS23 confers rice drought tolerance [39], which urges us to examine whether OsMADS23 or SAPK9 acts a substrate of OsPUB16. We first tested whether OsPUB16 interacts with OsMADS23 in the Y2H assay, with a subset of other rice U-box E3 proteins as control. The result showed that OsPUB16, but not other U-box proteins, specifically interacts with OsMADS23 (Figs 6A and S7A). Notably, OsPUB16 does not interact with SAPK9 in the Y2H assay (S7B Fig). Then, the GST pull-down assay showed that GST-OsPUB16, but not GST alone, pulled down His-OsMADS23 (Fig 6B), indicating that OsPUB16 physically interacts with OsMADS23 *in vitro*. The interaction of OsPUB16-OsMADS23 was further verified by BiFC, illustrated by the strong YFP fluorescence signal in the epidermal cells of *N*. *benthamiana* leaves harboring both OsPUB16-cYFP and OsMADS23-nYFP (Fig 6C). Consistently, the LCI assay also confirmed the interaction of OsPUB16 with OsMADS23 (Fig 6D). These results demonstrate that OsPUB16 interacts with OsMADS23 *in vivo* and *in vitro*, suggesting OsMADS23 might act as one of the substrates of OsPUB16.

We then tested whether OsPUB16 could ubiquitinate OsMADS23 by using a semi-endogenous ubiquitination assay. Total protein extracts from WT or OsPUB16-OX plants were incubated with GST-OsMADS23, in the presence of MG132, and then the immunoprecipitation was performed with anti-GST beads. We used anti-Ubi antibody to detect the immunoprecipitated GST-OsMADS23 and found that the abundance of poly-ubiquitinated GST-OsMADS23 was increased in OsPUB16-OX plant extracts (Fig 6E), indicating that OsPUB16 could mediate OsMADS23 ubiquitination. A close link between phosphorylation and ubiquitination has been implicated, and the E3 ligase-substrate association is regulated by phosphorylation [34]. Thr-20 and Ser-36 in OsMADS23 are main phosphorylation sites recognized by SAPK9 [39]. We next checked whether phosphorylation status of OsMADS23 affects its ubiquitination level. Interestingly, we found that the mimicked phosphorylation markedly blocked ubiquitination of the protein when GST-OsMADS23^T20D S36D^ was incubated with protein extracts from OsPUB16-OX plants (Fig 6E). Then, we investigated whether the phosphorylation status of OsMADS23 influences its interaction with OsPUB16, and thus affects its ubiquitination level. Expectedly, our GST pull-down assay showed that, compared to native GST-OsMADS23, the mimicked dephosphorylation (GST-OsMADS23^T20A S36A^) strengthened its interaction with His-OsPUB16, while the mimicked phosphorylation (GST-OsMADS23^T20D S36D^) reduced their interaction (Fig 6F). These results demonstrate that OsPUB16 targets OsMADS23 for ubiquitination, which is regulated by the phosphorylation status of OsMADS23.

Next, we investigated the influence of OsPUB16 on the stability of OsMADS23 by the cell-free degradation assay. GST-OsMADS23 protein was incubated with equal amount of protein extracts from WT or *ospub16-1* mutant. Immunoblot analysis showed that GST-OsMADS23 degradation was much slower in the extracts from *ospub16-1* mutant than that in WT (Fig 6G and 6H), manifesting that OsPUB16 mediates OsMADS23 degradation. ABA-induced phosphorylation of OsMADS23 mediated by SAPK9 is critical for OsMADS23 stability [39]. We found that the degradation of GST-OsMADS23^T20A S36A^ mediated by OsPUB16 was much faster, while degradation of GST-OsMADS23^T20D S36D^ was slower than its native form (Fig 6G and 6I), suggesting that phosphorylation increased OsMADS23 stability. These data demonstrate that OsPUB16 meditates the ubiquitination and degradation of OsMADS23 in plants, which is controlled by its phosphorylation status. These observations also confirm the close link between the two types of post-translational modifications, phosphorylation and ubiquitination.

### OsMADS23 promotes the JA response

**Fig 7:**
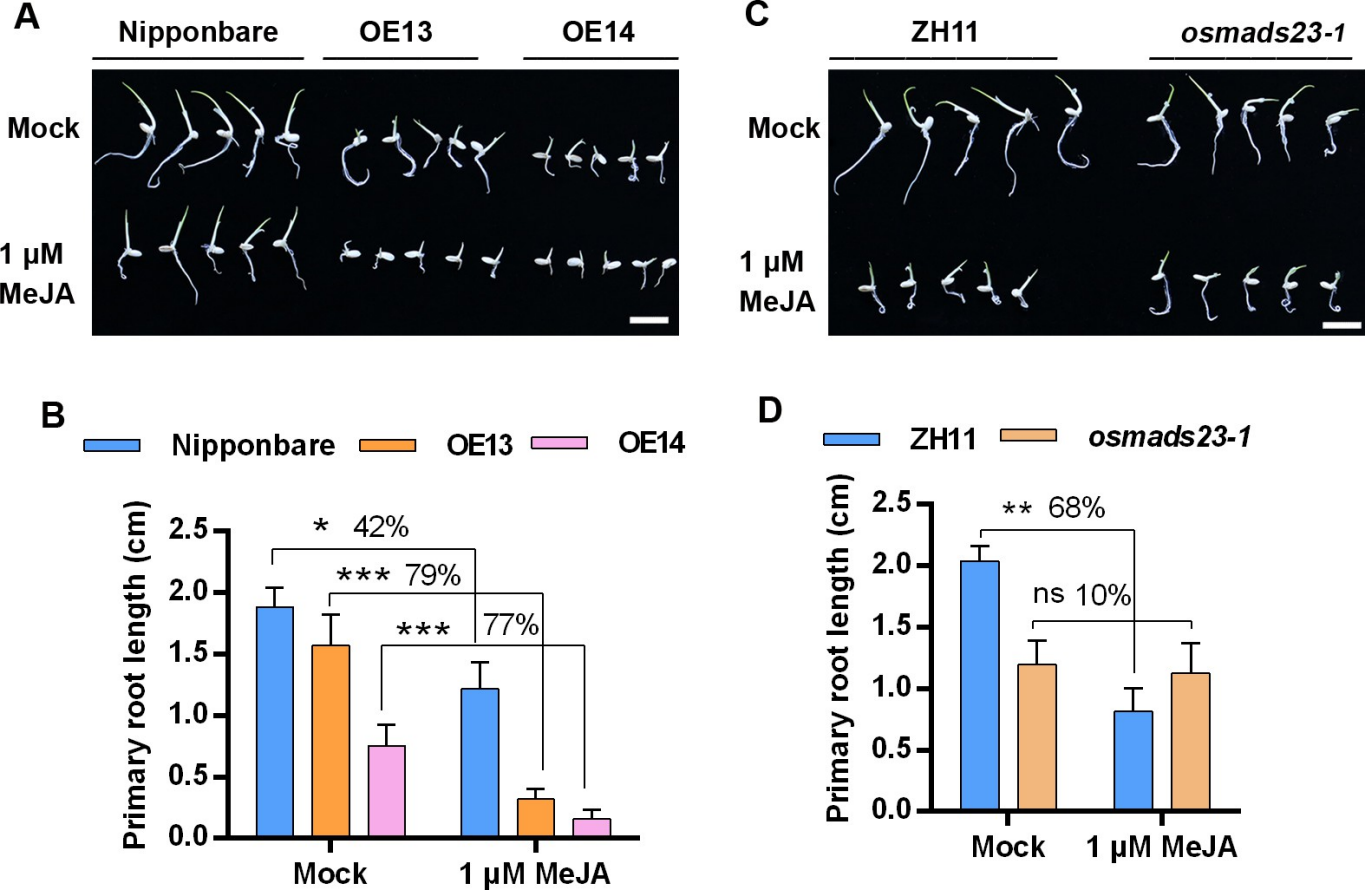
OsMADS23 mediates JA sensitivity indicated by primary root growth inhibition assay. **(A,C)** Images showing the responses of the wild type (Nipponbare) and *OsMADS23* overexpression lines (A), or wild type (ZH11) and *osmads23-1* mutant (C) to exogenous MeJA. Scale bars, 1 cm. Uniformly germinated seeds were grown on 1/2MS medium with or without MeJA for 2 days. **(B, D)** Statistical analysis of the primary root length of indicated plants in (A) and (C), respectively. Data are means ± SD (*n* = 15). In (B) and (D), the significant difference between the treated and untreated plants was determined by Student’s *t* test. *p < 0.05, **p < 0.01 or ***p < 0.001. ns, not significant.

The interaction of OsPUB16 with OsMADS23 as well as JAZ proteins urges us to examine whether OsMADS23 interacts with these JAZ proteins. Unexpectedly, the result of Y2H assay showed that OsMADS23 does not interact with this subset of JAZ proteins (S8 Fig). Given that OsPUB16 modulates JA and ABA response (Fig 4), and OsMADS23 regulates ABA response [39], we queried whether OsMADS23 also regulates JA response. Next, we evaluated the JA sensitivity of the loss-of-function mutant *osmads23-1*, *OsMADS23*-overexpressing lines (OE13, OE14) and their corresponding wild type through the primary root elongation inhibition assay. We found that, similar to their response to exogenous ABA [39], *OsMADS23*-overexpressing lines were more sensitive but *osmads23-1* mutant was more tolerant to MeJA than their corresponding wild type (Fig 7), indicating that overexpression of *OsMADS23* increases plant JA response. These data suggest a positive regulatory role of OsMADS23 in the interplay between ABA and JA signaling pathways.

### OsMADS23 directly activates *OsAOC* transcription by binding to its promoter

**Fig 8:**
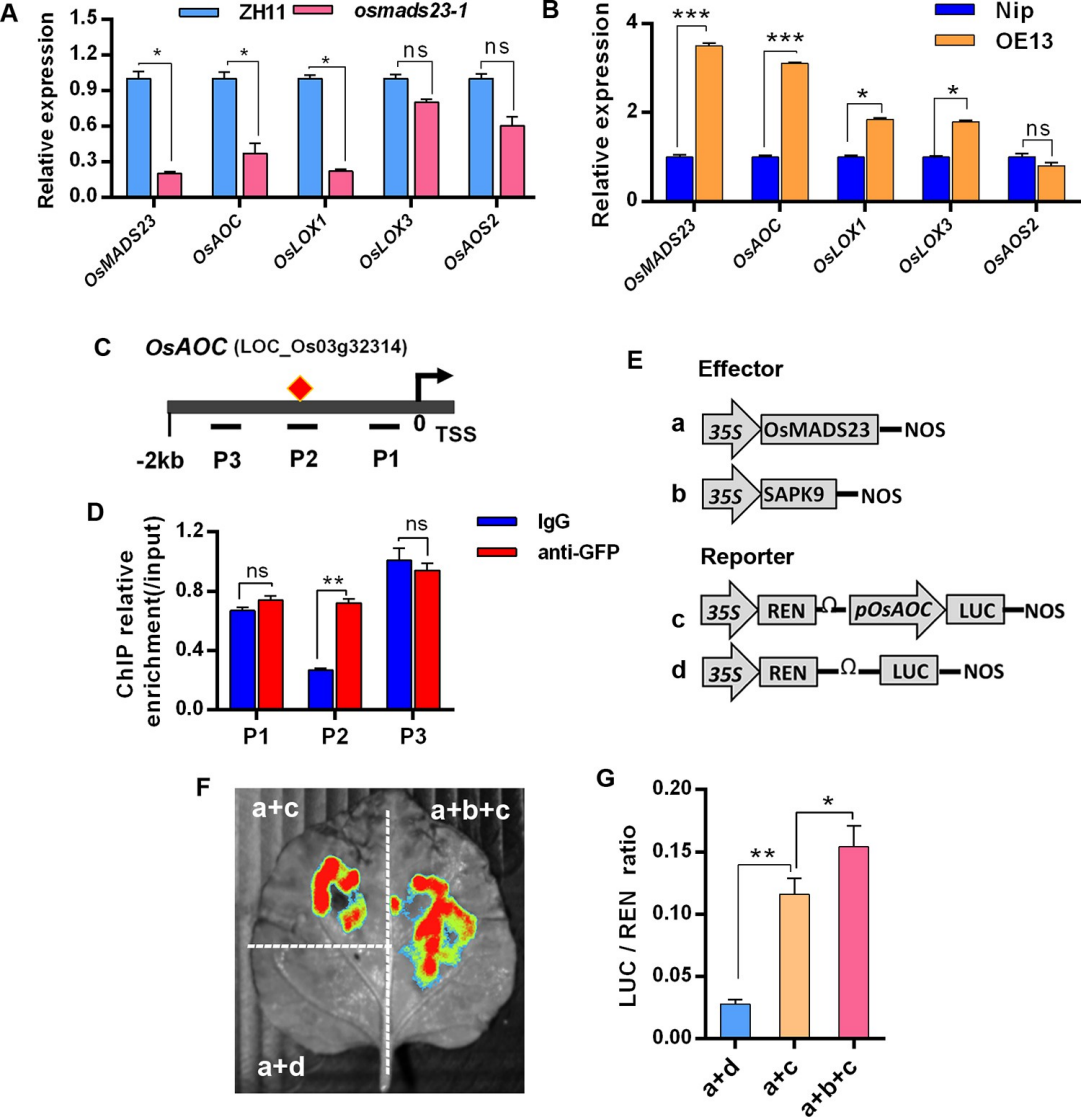
OsMADS23 directly regulates *OsAOC* transcription. **(A, B)** Expression of JA-biosynthetic genes in wild type (ZH11) and *osmads23-1* mutant (A), wild type (Nipponbare) and *OsMADS23* overexpression line (OE13) (B). Data are means ± SD (*n* = 3 biological replicates). *OsActin1* gene was used as internal control. **(C)** Schematic diagram of *OsAOC* promoter region (2 kb fragment upstream from transcription start site) showing the positions of CArC-box. P1-P3 fragments were used for amplification by ChIP-qPCR analysis. **(D)** ChIP-qPCR analysis of *OsAOC* promoter fragments enriched by OsMADS23-GFP with anti-GFP antibody in OsMADS23-GFP plants. Data are means ± SD (*n* = 3). P1-P3 represents the regions shown in (C). **(E)** Schematic diagrams of the effector and reporter used for transient transactivation assays. The fragment from -709 to -1184 bp in the promoter of *OsAOC* (*pAOC*) was used for constructing reporter. **(F, G)** Transactivation activity of OsMADS23 was enhanced by SAPK9, indicated by the LUC fluorescence imaging and LUC/REN ratio, respectively. Data are means ± SD (*n* = 3). The transient transactivation activity assays were performed in the leaves of *Nicotiana benthamiana* by agroinfiltration expression system. In (A), (B), (D), and (G), the significant difference was determined by Student’s *t* test. *p < 0.05, **p < 0.01 or ***p < 0.001. ns, not significant.

Considering the fact that OsMADS23 positively regulates JA response, we then examined the transcription levels of a couple of JA-biosynthetic genes in *osmads23-1* mutant, *OsMADS23*-overexpressing lines and wild type. Intriguingly, the expression of JA biosynthesis step-limiting genes *OsAOC* and *OsLOX1* [47,48] was significantly downregulated in *osmads23-1*, but upregulated in *OsMADS23*-overexpressing plants (Fig 8A and 8B), suggesting that *OsAOC* and *OsLOX1* are likely to be the downstream targets of OsMADS23. MADS-box transcription factors regulate gene transcription through binding to the elements containing CArG-box in the promoter region of target genes [49]. One CArG-box *cis*-element was found in 2 kb-promoter region of *OsAOC* (Fig 8C). Thus, we performed ChIP-qPCR assays in OsMADS23-GFP plants to test whether OsMADS23 binds to the promoter region of *OsAOC in vivo*. Substantial DNA enrichment was observed in the *OsAOC* promoter region containing CArG-box, while little enrichment was found in other regions (Fig 8D), hinting that OsMADS23 directly regulates the transcription of *OsAOC*, the master regulator of JA biosynthesis in the production of JA precursor 12-oxophytodienoic acid (12-OPDA). To further verify the regulation of *OsAOC* by OsMADS23, we performed a transient transactivation assay in *N*. *benthamiana* leaves through the luciferase reporter assay. The promoter region of *OsAOC* (*pOsAOC*) was fused to the *LUC* gene to generate the reporter, while the effector construct contained *OsMADS23* driven by the *CaMV 35S* promoter (Fig 8E). In support of the ChIP-qPCR result, the LUC/REN ratio as well as the luciferase fluorescence signal intensity was remarkably increased in *N*. *benthamiana* leaves co-expressing the reporter carrying the *OsAOC* promoter driving *LUC* with the effector containing OsMADS23, in comparison with the negative control (Fig 8F and 8G), demonstrating that OsMADS23 significantly induces the expression of *LUC* driven by *OsAOC* promoter. Further, to examine the impact of ABA signaling on *OsAOC* transcription regulated by OsMADS23, OsMADS23 was co-expressed with SAPK9 in *N*. *benthamiana* leaves as effectors. Notably, the expression of *LUC* was significantly upregulated in leaves co-expressing OsMADS23 with SAPK9 as effectors, compared to expressing OsMADS23 alone (Fig 8F and 8G). Thus, we conclude that OsMADS23 is an upstream transcriptional activator of *OsAOC* and regulates its expression *in vivo*, and SAPK9 enhances the transactivation activity of OsMADS23, and thus elevates *OsAOC* expression. Here, we identified a ‘SAPK9-OsMADS23-*OsAOC*’ pathway, through which ABA promotes JA biosynthesis.

### SAPK9 interferes with OsPUB16-OsMADS23 interaction through the phosphorylation on OsMADS23

**Fig 9:**
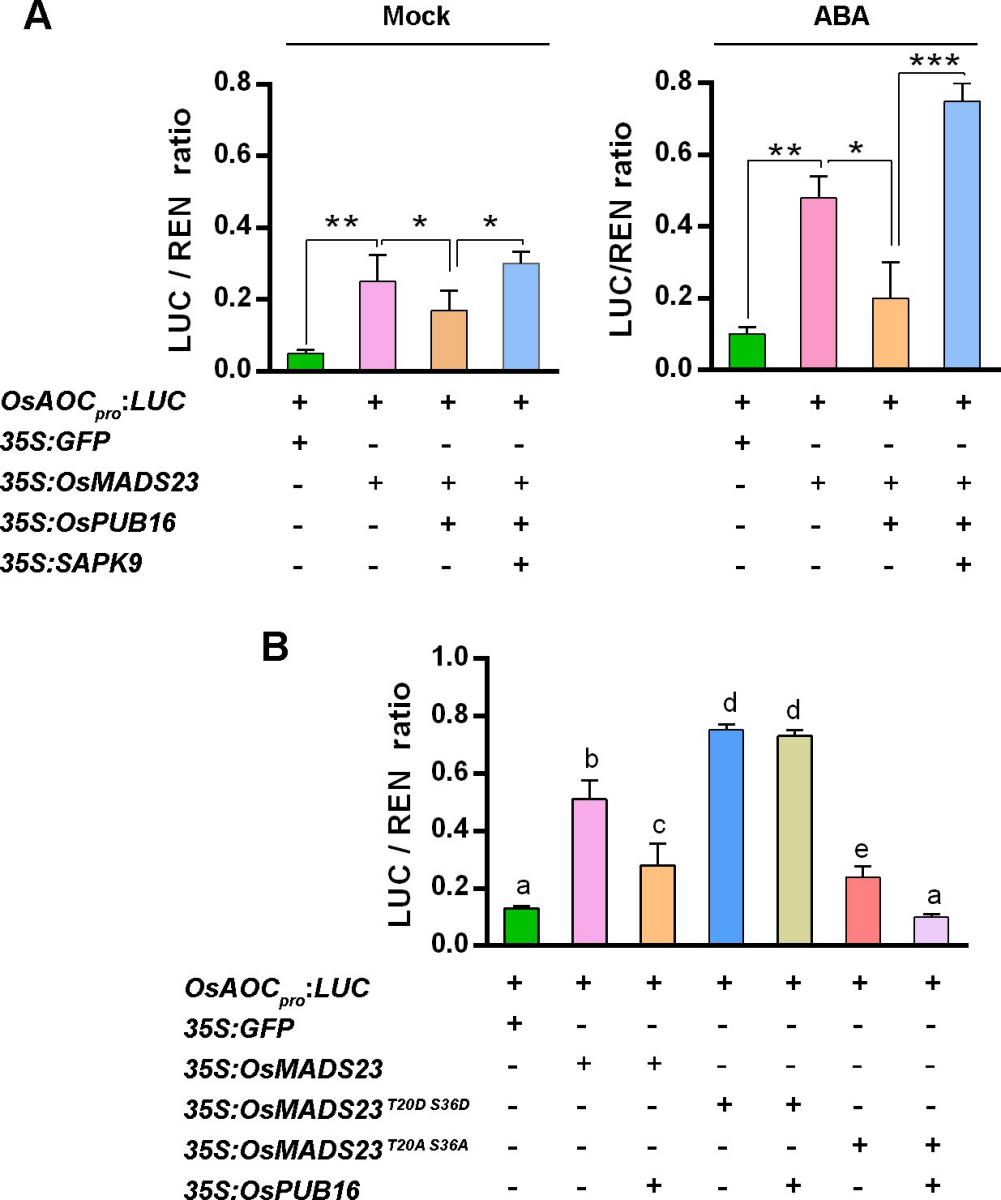
The influence of SAPK9 or OsPUB16 on the transcriptional activity of OsMADS23. **(A)** Luciferase transient transcriptional activity showing the influence of SAPK9, OsPUB16 or ABA on the transcriptional activity of OsMADS23. Luciferase transient transcriptional activity assay was performed in *Nicotiana benthamiana* leaves co-transfected with the reporter and different effector constructs. The transfected leaves treated with and without 50 μM ABA for 2 hours were used. Data are means ± SD (*n* = 3). The significant difference was determined by Student’s *t* test. *p < 0.05, **p < 0.01 or ***p < 0.001. ns, not significant. **(B)** Luciferase transient transcriptional activity showing OsMADS23 phosphorylation reducing the repression of its transcriptional activity mediated by OsPUB16. Data are means ± SD (*n* = 3). Recombinant GST-OsMADS23 or its different mutated versions (GST-OsMADS23^T20A S36A^ and GST-OsMADS23^T20D S36D^) was used. Two-way ANOVA followed by Bonferroni’s post-hoc test was performed. Different letters with the same superscript mark indicate significant differences (*p* < 0.05). The transient transactivation activity assays were performed in the leaves of *N*. *benthamiana* by agroinfiltration expression system.

Our previous findings showed that SAPK9 interacts with OsMADS23, resulting in the increased stability of OsMADS23 via phosphorylation [39]. Our current observation showed OsPUB16 interacts with OsMADS23 to promote its ubiquitination and degradation (Fig 6), but OsPUB16 does not physically interact with SAPK9 (S7B Fig). These observations suggest that SAPK9 might interfere with the OsPUB16-OsMADS23 interaction via the phosphorylation on OsMADS23. To verify our hypothesis, we co-transformed OsPUB16-nYFP and OsMADS23-cYFP with or without SAPK9 into *N*. *benthamiana* leaves and analyzed the OsPUB16-OsMADS23 interaction by confocal microscopy. The result showed that, compared to co-expressing OsPUB16-nYFP and OsMADS23-cYFP alone, the fluorescent signal drastically decreased when co-expressing OsPUB16-nYFP and OsMADS23-cYFP with SAPK9-FLAG (S9 Fig), suggesting that SAPK9 interferes with the OsPUB16-OsMADS23 interaction. Further, ABA treatment markedly enhanced the interference effect of SAPK9 on the OsPUB16-OsMADS23 interaction (S9 Fig). We then examined the effect of SAPK9 on OsPUB16-OsMADS23 interaction by investigating OsMADS23 transcription regulation of the downstream target gene through transient transactivation assays. Compared to OsMADS23 alone, co-expression of OsPUB16 with OsMADS23 repressed *LUC* expression driven by the *OsAOC* promoter, while the LUC/REN ratio was enhanced when SAPK9 was added (Fig 9A), confirming the interference of SAPK9 on OsPUB16-OsMADS23 interaction. Notably, after ABA treatment, SAPK9 drastically increased *LUC* expression (Fig 9A), implying that SAPK9-mediated phosphorylation on OsMADS23 interferes OsPUB16-OsMADS23 interaction, and thus enhances OsMADS23 stability. This observation was further verified by using the mutated forms of OsMADS23 protein. Different from co-expression of OsPUB16 with native OsMADS23 repressing *LUC* expression, the transactivation activity of OsMADS23^T20D S36D^ was not greatly reduced by OsPUB16, while the LUC/REN ratio from OsMADS23^T20A S36A^ significantly decreased when OsPUB16 was added (Fig 9B). These results demonstrate that OsMADS23 phosphorylation reduces its ubiquitination level, and thus promotes its stability, indicated by its transactivation activity.

## Discussion

Plants tightly control intracellular protein levels via various strategies to adapt to various adverse conditions. Emerging evidence indicates that the E3 ubiquitin ligase-mediated degradation pathway is one of the most important mechanisms maintaining the homeostasis of intracellular proteins [50]. The U-box E3 ubiquitin ligases are widely present in eukaryotic organisms. Arabidopsis, a dicot model plant, contains 64 U-box E3 ubiquitin ligases, whereas the monocot model crop rice harbors 77 U-box proteins [51,52]. To date, A few U-box E3 ubiquitin ligases have been identified to be involved in plant environmental stress responses by modulating ABA signaling [33,35,38]. Whether E3 ubiquitin ligases modulate plant stress response via the interplay between ABA and other phytohormone signaling pathways still needs to be elucidated. In our study, we investigated how OsPUB16 integrates ABA and JA signaling pathways to regulate plant water-deficit response in rice by using CRISPR/Cas9-mediated knockout mutant *ospub16-1* and *ospub16-2* (+/-). *ospub16-1* could grow, but *ospub16-2* (-/-) was lethal. As to this, there might be two reasons. The large DNA fragment deletion in *ospub16-2* genome caused earlier termination of protein translation than that in *ospub16-1*, which might be responsible for the lethality of the *ospub16-2* mutant. It has been reported that, in addition to causing frameshift mutations, CRISPR/Cas9-mediated sequence alterations in the coding region of the target gene may also result in altered splicing of the respective pre-mRNA, and such altered splicing products also give rise to aberrant protein [53–55]. In the *ospub16-1* mutant, some kind of functional mRNA of *OsPUB16* might be expressed, perhaps by the altered splicing, which ensures its survival.

### OsPUB16 negatively regulates plant water-deficit tolerance by modulating the ‘SAPK9-OsMADS23-*OsAOC*’ pathway

Crosstalk among phytohormones is crucial for plant growth as well as adjustment to various environments, and previous studies have provided the evidence that ABA interacts with JA signaling to synergistically regulate plant multiple physiological processes [17,20,22]. In this current study, our findings demonstrate that OsPUB16 negatively modulates rice water-deficit tolerance by repressing ABA and JA biosynthesis (Figs 2–4). The increased ABA/JA sensitivity and ABA/JA levels in *ospub16* mutants (Fig 4) urge us to ask a critical question whether OsPUB16 modulates the interplay between ABA and JA signaling pathways. If it is, how does it achieve? Here, our findings provide the convincing evidence that OsPUB16 regulates the link between JA and ABA signaling pathways during plant water-deficit response. First, biochemical evidence demonstrated OsPUB16 interacts with a subset of JA signaling repressors (Fig 5A–5D); meanwhile, OsPUB16 interacts with OsMADS23 (Fig 6A–6D), a transcription factor that promotes ABA signaling by activating ABA-biosynthetic genes *OsNCEDs* [39]. These physical interactions reveal a direct link between JA and ABA signaling at the molecular level. Second, OsMADS23 acts as an upstream activator to regulate the transcription of JA-biosynthetic gene *OsAOC*, and SAPK9-mediated phosphorylation on OsMADS23 increases its transcription activity (Fig 8E–8G). Finally, ABA-induced SAPK9-mediated phosphorylation on OsMADS23 interferes with the OsPUB16-OsMADS23 interaction (S9 Fig), which reduces OsMADS23 ubiquitination and degradation and therefore enhances its regulatory effect on *OsAOC* (Fig 9). Therefore, our results strongly demonstrate that OsPUB16 represses ABA and JA biosynthesis by regulating the module ‘SAPK9-OsMADS23-*OsAOC*’.

Previous efforts have provided clues that MYC2 as well as JAM1 might serve as the linkers of ABA and JA signaling pathways [18,56], and recent studies have shown that the ABA promotes JA biosynthesis through a novel pathway ‘SAPK10-bZIP72-*OsAOC*’ [22]. Nevertheless, the exact mechanisms underlying the link between ABA and JA signaling still remain to be elucidated, particularly during plant water-deficit response. Here, our study provides the new evidence of the link between JA and ABA signaling. In the ‘SAPK9-OsMADS23-*OsAOC*’ pathway, ABA promotes the JA biosynthesis to synergistically promote plant water-deficit tolerance.

As key suppressors in JA signaling, JAZ proteins modulate JA signaling through a negative regulatory feedback loop involving MYC2, suggesting a mechanism for the rapid switching on and off of JA pathway in response to a JA pulse [57,58]. In our study, characterization of the physical interactions between OsPUB16 and a subset of JAZ proteins (Fig 5A) may shed light on the molecular basis of the regulation of the JA signaling by U-box E3 ubiquitin ligases. Interestingly, OsPUB16 mediates the ubiquitination and subsequent degradation of JAZ proteins in a JA-dependent manner (Fig 5E–5G), suggesting that OsPUB16 is likely to promote JA response; on the other hand, OsPUB16 represses JA response by inhibiting JA biosynthesis (Fig 4E–4H and 4J). These observations seem to be contradictive, but actually inflect the negative regulatory feedback on the increased JA response. How OsPUB16 finely tunes the JA response switching on and off in the changing environment is a challenging but interesting research, which needs to be explored in the future work.

### The phosphorylation status of OsMADS23 modulates its ubiquitination level and stability

The current study elaborates a key module ‘SAPK9-OsMADS23-*OsAOC*’ regulated by OsPUB16 during plant water-deficit response. In the module, there is a connection between the phosphorylation and ubiquitination of OsMADS23, which acts a central regulator to determine plant water-deficit response. Our findings showed that, compared to the native form, the phosphorylated OsMADS23 (OsMADS23^T20D S36D^) reduced its interaction with OsPUB16, and thus was less ubiquitinated by OsPUB16 (Fig 6E and 6F), demonstrating that phosphorylation of OsMADS23 regulates its ubiquitination. Consistently, phosphorylated OsMADS23 (OsMADS23^T20D S36D^) was more stable, while the dephosphorylated OsMADS23 (OsMADS23^T20A S36A^) was labile than the native form (Fig 6G–6I). This observation was further confirmed by the fact that OsMADS23^T20D S36D^ had increased transactivation activity, compared to its native form (Fig 9B). OsPUB16 reduces the ABA and JA biosynthesis via mediating OsMADS23 ubiquitination and degradation, whereas SAPK9 enhances the ABA and JA signaling via mediating OsMADS23 phosphorylation and stabilization. Although ‘SAPK9-OsMADS23-*OsAOC*’ is identified as a pathway that links ABA and JA signaling pathways, how OsPUB16 precisely regulates the balance between plant growth and environmental adaption through the module still needs to be revealed.

**Fig 10:**
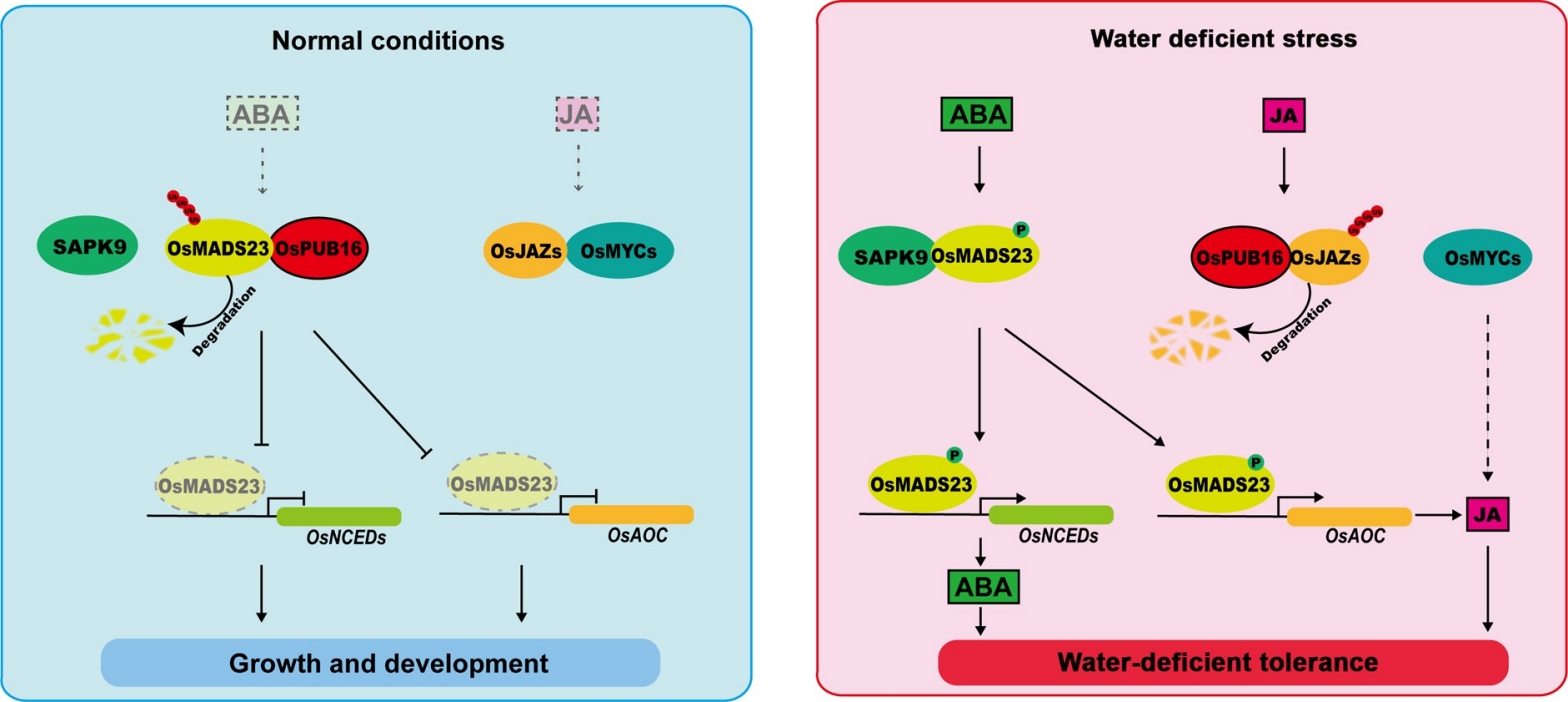
A proposed working model for OsPUB16 modulating water-deficit response in rice. Under normal growth conditions, OsPUB16 interacts with OsMADS23 and mediates its ubiquitination and degradation, therefore repressing the expression of *OsAOC* and *OsNCED*s and ABA/JA biosynthesis (left); when plants are subjected to water deficit stress, rapid ABA accumulation activates SAPK9. SAPK9-mediated phosphorylation on OsMADS23 interferes with the OsPUB16-OsMADS23 interaction, resulting in OsMADS23 stabilization. Phosphorylated OsMADS23 activates ABA- and JA-biosynthetic gene expression and thus promoting ABA- and JA-mediated drought tolerance (right).

Based on our results, we propose a model to elucidate how OsPUB16 interacts with its partners to modulate the water-deficit response in rice (Fig 10). Under normal growth conditions, SAPK9 is deactivated, and therefore OsPUB16 interacts with OsMADS23, resulting in the OsMADS23 ubiquitination and degradation through the 26*S* proteasome system, and thus repressing of JA- and ABA-biosynthetic gene expression (Fig 10, left). Under water deficit conditions, rapid ABA accumulation activates SAPK9, which then interferes with OsPUB16-OsMADS23 interaction via the phosphorylation on OsMADS23, leading to enhanced OsMADS23 stability and subsequent ABA and JA biosynthesis, thus promoting ABA- and JA-mediated water-deficit tolerance (Fig 10, right).

## Materials and methods

### Plant materials and growth conditions

Rice (*Oryza sativa* ssp. *japonica*) *cv* Nipponbare was used in this study. The knockout mutants of *OsPUB16* were generated by CRISPR-Cas9 system from Nipponbare. For generating *OsPUB16*-overexpressing lines, the open reading frame (ORF) of *OsPUB16* fusion with *GFP* (*green fluorescence protein*) driven under the *35S* promoter was cloned into pCAMBIA1301, and then transformed into rice calli by *Agrobacterium tumefaciens*-mediated transformation [59]. For the complementation test, the *OsPUB16* coding sequence driven by its promoter region (2,509 bp) was cloned into the binary vector pCAMBIA1301, and the resulting construct was introduced into *ospub16-1* mutant. The seeds of homozygous transgenic plants were used for the research. T-DNA insertion mutants of *OsMADS23* and its overexpression lines were used [39]. Plants were grown in the growth chamber or greenhouse with a 14-hour light (30°C) /10-h dark (25°C) cycle (300 μmol photons m^-2^s^-1^) with 60% humidity. Primers for vector construction and transformant identification are listed in S1 Table.

### Phytohormone sensitivity assessment

For seed germination inhibition assay, sterilized seeds were sown on 1/2MS medium supplemented with different concentration of ABA or MeJA at 28°C with a 12-hour photoperiod for germination. Germinated seeds were counted every day. Complete germination was defined as the growth of the coleoptile to a length of 5 mm. For the primary root elongation inhibition assay, uniformly germinated seeds were grown on 1/2MS medium supplemented with 3 μM ABA or 2 μM MeJA for 3 days at 28°C, and the primary root length of these seeding were measured.

### Water-deficit tolerance and water loss assays

To evaluate the water-deficit tolerance of different genotypes, plants were grown in pots filled with equal amount of soil and cultivated under normal growth conditions for 3 weeks, with equal irrigation. Then, these plants were exposed to the same severity of soil drying by withholding water for 12 days under the same growth environment. Survival rates were calculated after 5 days of rewatering. For the water loss assay of leaf, the detached leaves from 2-month-old plants were placed at room temperature under dim light, and the fresh weight of leaves was monitored at the indicated time points. Water loss rate (WLR) was calculated from the decrease in weight at indicated time points compared with time zero, based on the following formula WLR (%) = (initial weight–final weight) / initial weight × 100%. The average survival rate and water loss rate were calculated from three independent experiments.

### Stomatal observation

The stomatal movement observation was performed as described previously [39]. Two-week-old seedlings were subjected to light for at least 2 hours to ensure stomata open. Then the fully expended young leaves were floated in the buffer (1 mM MES-NaOH, 20 mM KCl, pH 6.0) with 0 or 30 μM ABA for 3 hours. After treatment, the samples were frozen in liquid nitrogen immediately. Then the stomata were detected with a Hitachi TM3000 scanning electron microscope with a -35°C cold stage. At least 200 stomata from 5 independent plants were observed in each treatment of each line. Three independent experiments were repeated.

### RNA extraction and quantitative real-time PCR (qRT-PCR) analysis

Total RNA was extracted from 2-week-old seedlings using TRIzol reagent (Takara). The qRT-PCR analysis was performed with *OsActin1* as the internal control. Relative changes in gene expression level were quantified based on three biological replicates via the 2^-ΔΔCt^ method [60]. Primers used for expression analysis are given in S1 Table. Three independent experiments were repeated.

### Subcellular localization assay

The ORF of *OsPUB16* fused with *GFP* was cloned into pCAMBIA1301 (for primers, in S1 Table), and the construct was transformed into 4-week-old *Nicotiana benthamiana* leaves with an efficient agroinfiltration expression system [61]. After infiltration, *N*. *benthamiana* grew at 25°C for 48–72 hours, and the leaf epidermis was used for detecting GFP fluorescence signals under confocal laser scanning microscope (Leica SP8).

### Yeast two-hybrid assay

We used the Matchmaker Gold Yeast Two-Hybrid System (Clontech) to perform the yeast two-hybrid assay, according to the manufacturer’s instructions. The ORF of *OsPUB16* was cloned into pGBKT7 vector, and *JAZ*s or *OsMADS23* was cloned into pGADT7 vector, respectively. Then the corresponding plasmids were co-transformed into the yeast strain Y2HGold for two-hybrid assay. The transfected yeast cells were plated on synthetic defined (SD)/-Leu-Trp (-LW) or SD/-Ade-His-Leu-Trp (-LWHA) medium, and incubated at 28°C for 2 days. Primers for these constructs are listed in S1 Table.

### Bimolecular fluorescence complementation (BiFC) assay

BiFC vectors pFGC-nYFP and pFGC-cYFP [62] were used. The ORF of *OsPUB16* was cloned into pFGC-cYFP and *OsJAZ9* or *OsMADS23* was cloned into pFGC-nYFP. The generated cYFP and nYFP constructs were transformed into *Agrobacterium* GV3101, respectively, and then co-infiltrated into 4-week-old *N*. *benthamiana* leaves with an efficient agroinfiltration expression system [61], with HY5-RFP as a nuclear-localized marker. After infiltration, *N*. *benthamiana* grew at 25°C for 48–72 hours, and YFP and RFP fluorescence signals were visualized using the confocal microscope (Leica SP8). The primers used for these constructs are listed in S1 Table.

### Firefly luciferase complementation imaging (LCI) assay

JW771 and JW772 vectors were used, and the LCI assay was performed in *N*. *benthamiana* leaves as described previously [63]. Briefly, the ORF of *OsPUB16* or *OsMADS23* was fused with the N- and C-terminal parts of the luciferase reporter gene *LUC* (nLUC and cLUC), respectively, to generate the constructs OsPUB16-nLUC and cLUC-OsMADS23. The resulting constructs were transformed into *N*. *benthamiana* leaves with the agroinfiltration expression system. After 36 hours, the leaves were sprayed with 0.32-mg/ml D-Luciferin potassium salt in 0.1% (v/v) Triton X-100, and the LUC signal was observed under low-light cooled CCD imaging apparatus (Alliance, UK). The primers used for BiFC assay are listed in S1 Table.

### GST pull-down assay

The ORF of *OsPUB16* was cloned into pGEX-4T-1 and transformed into DE3 to produce GST-OsPUB16, and *OsMADS23* or *OsJAZ9* into pET-28a to produce His-OsMADS23 or His-OsJAZ9 (for primers, in S1 Table). For the pull-down assay, GST-OsPUB16 or GST was incubated with GST Bind Resin overnight at 4°C for 2 hours, and then purified His-OsMADS23 or His-OsJAZ9 was added for incubation overnight. The beads were washed with pull-down buffer for 3 times, and then the bounded proteins were finally eluted. The pulled-down proteins were analyzed by immunoblot analysis with the anti-His antibody (Proteintech, 66005-1-Ig) and anti-GST antibody (Proteintech, 66001-2-Ig), respectively.

### Chromatin immunoprecipitation-quantitative PCR (ChIP-qPCR) analysis

The EpiQuik Plant ChIP Kit (Epigentek) was used for ChIP assays. Briefly, 10-day-old OsMADS23-GFP seedlings were harvested and fixed in 1% formaldehyde, and chromatin was isolated from 2 *g* crosslinked leaves. Isolated chromatin was sonicated for DNA fragmentation ranging from 200 to 500 bp. Subsequently, the DNA/protein complex was immunoprecipitated with anti-GFP antibody (Abmart, M20004) or IgG. Then the immunoprecipitated DNA was purified with phenol/chloroform after reverse crosslinking and proteinase K treatment. The immunoprecipitated DNA was used for qPCR analysis. The primers for ChIP-qPCR used are listed in S1 Table.

### Semi-endogenous ubiquitination assay

Briefly, the total proteins were extracted from 10-day-old wild type or OsPUB16-OX plants by using the NP40 lysis buffer (Beyotime Biotechnology, P0013F). Then the protein extracts were incubated with GST-OsMADS23, GST-OsMADS23^T20D S36D^ or His-OsJAZ9 for 5 hours, in the presence of 50 μM MG132, and then the immunoprecipitation was performed with anti-GST or anti-His beads. The immunoprecipitated proteins were detected with anti-Ubi antibody (Santa Cruz Biotechnology, sc-8017) and anti-GFP antibody, respectively. The immunoblot analysis of Histone H3.1 (H3.1) with anti-H3.1 antibody (Solarbio, K800007P) was used as a loading control of protein lysates. Three independent experiments were repeated, and representative pictures were shown.

### Cell-free degradation assay

Cell-free degradation assays were performed as previously described [64]. Two-week-old seedlings were used to extract protein in the buffer (25 mM Tris-HCl, pH 7.5, 10 mM NaCl, 10 mM MgCl_2_, 5 mM DTT). Purified recombinant GST-OsMADS23, OsMADS23^T20D S36D^, OsMADS23^T20A S36A^ or His-OsJAZ9 was incubated with equal amount of protein extracts for different time, in the presence of 10 mM ATP. Anti-His antibody (Proteintech, 66005-1-Ig) or anti-GST antibody (Proteintech, 66001-2-Ig) were used to detect recombinant protein level by immunoblot analysis.

### Accession numbers

*OsPUB16* (LOC_Os01g66130), *OsMADS23* (LOC_Os08g33488), *SAPK9* (LOC_Os12g39630), *OsAOC* (LOC_Os03g32314), *OsLOX1* (LOC_Os03g49380), *OsAOS2* (LOC_Os03g12500), *OsLOX3* (LOC_Os03g49260), *OsJAZ5* (LOC_Os04g32480), *OsJAZ6* (LOC_Os03g28940), *OsJAZ7* (LOC_Os07g42370), *OsJAZ8* (LOC_Os09g26780), *OsJAZ9* (LOC_Os03g08310), *OsJAZ10* (LOC_Os03g08330), *OsJAZ11* (LOC_Os03g08320), *OsJAZ12* (LOC_Os10g25290), *OsJAZ13* (LOC_Os10g25230).

## Supporting information

S1 Fig Alignment of amino acid sequence of OsPUB16 with other U-box E3 ubiquitin ligases. **(A)** The phylogenetic analysis of a subset of PUB proteins from different plants. **(B)** Alignment of OsPUB16 with other plant U-box proteins. The solid red line indicates the conserved U-box domain, and solid block line indicates the ARM repeats domain. The accession numbers are as follows: OsPUB16 (LOC_Os01g66130.1), OsPUB15 (LOC_Os08g01900.1), SB03g379800 (Sobic.003G379800.1), SB07g011400 (Sobic.007G011400.2), ATPUB4 (AT2G23140.1), SlU-box9 (Solyc01g014230.2.1), SlU-box62 (Solyc12g100000.1.1), MDP000221363, MDP0000411998, StPUB40 (PGSC0003DMT400046296), GrPUB55 (Gorai.008G079700.1), GrPUB53 (Gorai.007G219900.1). **(C)** Schematic representation of the U-box (amino acids 269 to 332) domain and the ARM repeats near the C- terminus in OsPUB16 protein.
(TIF)

S2 Fig The spatio-temporal expression profile of *OsPUB16* analyzed using the PlaNet Browser. (TIF)

S3 Fig Morphological phenotypes of the *ospub16* mutants and *OsPUB16* overexpression lines. **(A)** Schematic presentation of the gene structure of *OsPUB16* and CRISPR-cas9 editing site. White boxes: untranslated regions; Black boxes: exons; black line: intron. TGG: PAM (protospacer adjacent motif). The CRISPR-cas9 target site and mutation were shown in homozygous mutants of *ospub16*. The letter in red represents the nucleotide insertion, and the red dots represent nucleotide deletion. **(B)** Frameshift mutations in *ospub16* mutants in (A) resulting in early termination of protein translation. **(C)** Two-week-old wild type (WT, Nip) and *ospub16* mutants. Scale bars, 2 cm. **(D)** Shoot length measurement for 2-week-old seedlings. Data are means ± SD (*n* = 10). **(E)** Three-month-old WT and *ospub16* mutants. Scale bars, 10 cm. **(F)** Plant height measurement for 3-month-old plants. Data are means ± SD (*n* = 10). **(G)** Analysis of *OsPUB16* by quantitative real-time PCR analysis. The transcript levels in wild type were defined as “1”. Error bars indicate SD (*n* = 3 biological replicates). *OsActin1* was used as the internal control. **(H)** Analysis of *OsPUB16* by semi-quantitative PCR analysis. The red arrows are the positions of primers for quantitative real-time PCR and semi-quantitative PCR analysis. **(I)** Four-month-old WT and *OsPUB16* overexpression (OX-4, OX-10) plants. Scale bar, 10 cm. **(J)** Expression analysis of *OsPUB16* independent *OsPUB16* overexpression transgenic lines (OX-4, OX-10). The transcript level in the wild type (Nip) was defined as “1”. Data are means ± SD (n = 3). **(K)** Plant height measurement for 4-month-old plants. Data are means ± SD (*n* = 10). In (D), (F), (G), (J) and (K), the significant difference between transgenic plants and wild type was determined by Student’s *t* test. *p < 0.05, **p < 0.01 or ***p < 0.001.
(TIF)

S4 Fig *OsPUB16* expression levels in WT, *ospub16-1* mutant and complementary lines (C-1, C-2). **(A)** Construction of the complementary vector. The coding sequence of *OsPUB16* driven by its own promoter was cloned into pCAMBIA1301. **(B)** Analysis of *OsPUB16* by quantitative real-time PCR analysis in WT, *ospub16-1* mutant and complementary lines. The transcript levels in WT were defined as “1”. Error bars indicate SD (*n* = 3 biological replicates). *OsActin1* was used as the internal control.
(TIF)

S5 Fig OsPUB16 mediates ABA and JA sensitivity indicted by seed germination inhibition. **(A, C)** Seed germination of WT and *ospub16-1* mutant on 1/2MS medium without or with different concentrations of ABA (A) or MeJA (B) at 4 DAG (day after germination). Bars = 1 cm. **(B, D)** Seed germination rate on 1/2MS medium without or with 5 μM ABA (J) or 3 μM MeJA (L) at 4 DAG. Data are means ± SD with biological triplicates (*n* = 3, each replicate containing 50 seeds). In (B) and (D), the significant difference between the treated and untreated plants was determined by Student’s *t* test. *p < 0.05.
(TIF)

S6 Fig The cell-free degradation assay for recombinant His-OsJAZ9 in the protein extracts from wild type (WT) and *ospub16-1* mutant. **(A)** Representative pictures showing the cell-free degradation assay for recombinant His-OsJAZ9, in the presence of 10 mM ATP. Recombinant His-OsJAZ9 was detected by anti-His antibody. The Coomassie blue–stained ribulose-1,5-bisphosphate carboxylase/oxygenase (Rubisco) large subunit (Rbc L) was used as a loading control. (B) Relative recombinant protein levels of His-OsJAZ9 at indicated time points in the cell-free degradation. Data are means ± SD (*n* = 3, three independent replicate experiments). The significant difference between the treated and untreated was determined by Student’s *t* test. ns, not significant.
(TIF)

S7 Fig **Yeast two-hybrid assays to test the interaction of OsPUB16 with OsMADS23 (A) or SAPK9 (B).** Yeast cells were grown on synthetic defined (SD)/-Leu-Trp (-LW) medium and SD/-Ade-His-Leu-Trp (-LWHA) medium. The AD-T + BD-P53 was used as a positive control, and AD-T + BD-lam as a negative control.
(TIF)

S8 Fig Yeast two-hybrid assays to test the interaction of OsMADS23 with rice JAZ proteins. Yeast cells were grown on synthetic defined (SD)/-Leu-Trp (-LW) medium and SD/-Ade-His-Leu-Trp (-LWHA) medium.
(TIF)

S9 Fig SAPK9 interferes with the OsPUB16-OsMADS23 interaction. Confocal images showing the interference SAPK9 with the OsPUB16-OsMADS23 interaction, particularly in the presence of ABA. OsPUB16-cYFP and OsMADS23-nYFP were co-expressed in the leaves of *Nicotiana benthamiana* with or without the presence of SAPK9. Strong YFP fluorescent was detected when OsPUB16-cYFP and OsMADS23-nYFP were co-expressed, but the fluorescent signals were reduced in the presence of SAPK9-FLAG, not FLAG alone. ABA treatment further reduced the YFP fluorescent signals in the leaves co-expressing OsPUB16-cYFP and OsMADS23-nYFP with SAPK9-FLAG, but not in the leaves containing OsPUB16-cYFP and OsMADS23-nYFP. HY5-RFP was used as a nuclear-localized marker. YFP and RFP fluorescence signals were visualized using the confocal microscope (Leica SP8). YFP, fluorescent channel in yellow and RFP fluorescent channel in red.
(TIF)

S1 Table List of primers. (XLSX)
