# Supplementary figures and images for: Rice OsPUB16 modulates the ‘SAPK9-OsMADS23-OsAOC’ pathway to reduce plant water-deficit tolerance by repressing ABA and JA biosynthesis
Source: PLoS Genet. 2022 Nov 28;18(11):e1010520. doi: 10.1371/journal.pgen.1010520 (PMC9731423; doi:10.1371/journal.pgen.1010520)

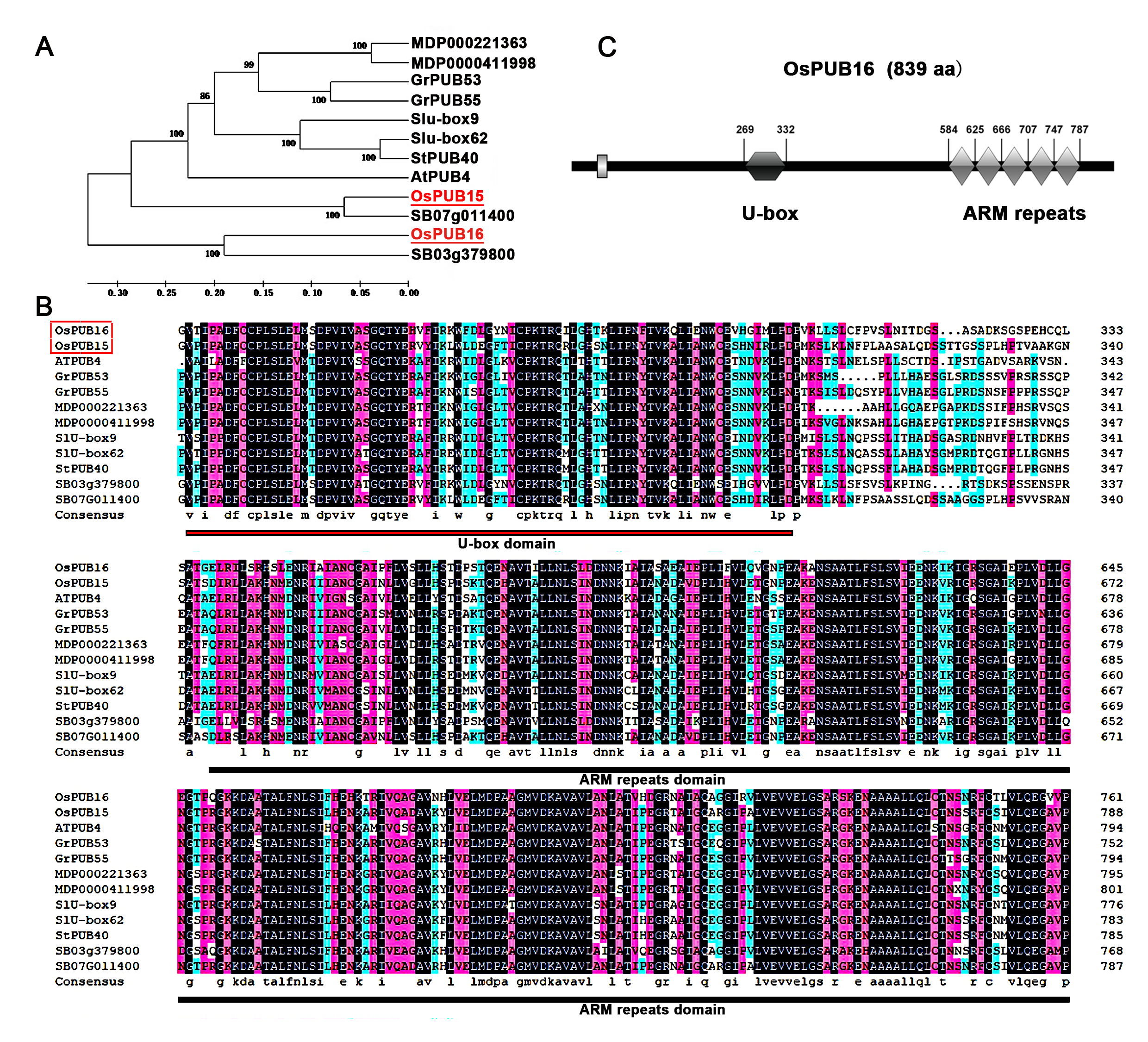

Supplement: S1 Fig — (A) The phylogenetic analysis of a subset of PUB proteins from different plants. (B) Alignment of OsPUB16 with other plant U-box proteins. The solid red line indicates the conserved U-box domain, and solid block line indicates the ARM repeats domain. The accession numbers are as follows: OsPUB16 (LOC_Os01g66130.1), OsPUB15 (LOC_Os08g01900.1), SB03g379800 (Sobic.003G379800.1), SB07g011400 (Sobic.007G011400.2), ATPUB4 (AT2G23140.1), SlU-box9 (Solyc01g014230.2.1), SlU-box62 (Solyc12g100000.1.1), MDP000221363, MDP0000411998, StPUB40 (PGSC0003DMT400046296), GrPUB55 (Gorai.008G079700.1), GrPUB53 (Gorai.007G219900.1). (C) Schematic representation of the U-box (amino acids 269 to 332) domain and the ARM repeats near the C- terminus in OsPUB16 protein. (TIF) [file pgen.1010520.s001.tif]

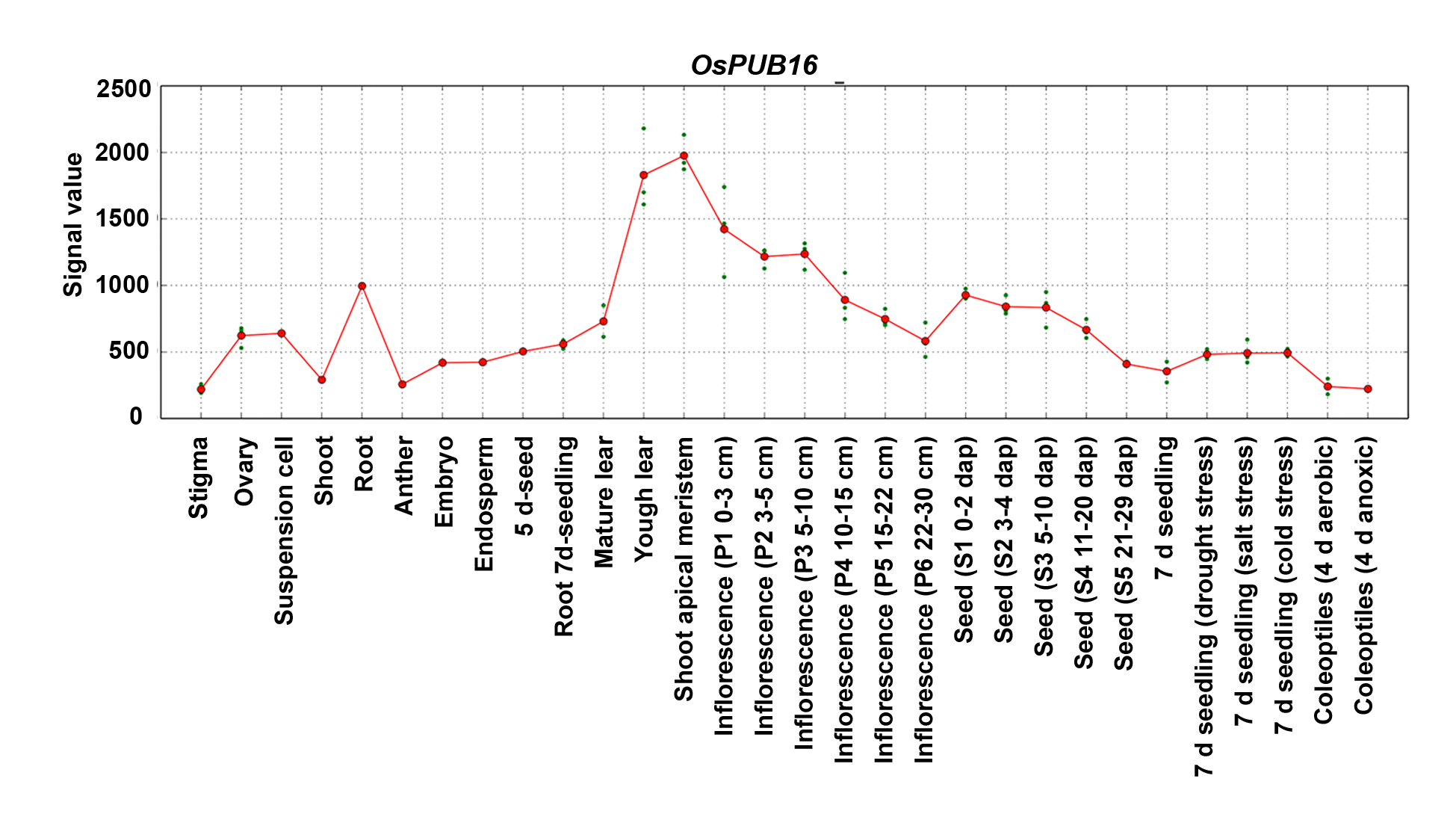

Supplement: S2 Fig — (TIF) [file pgen.1010520.s002.tif]

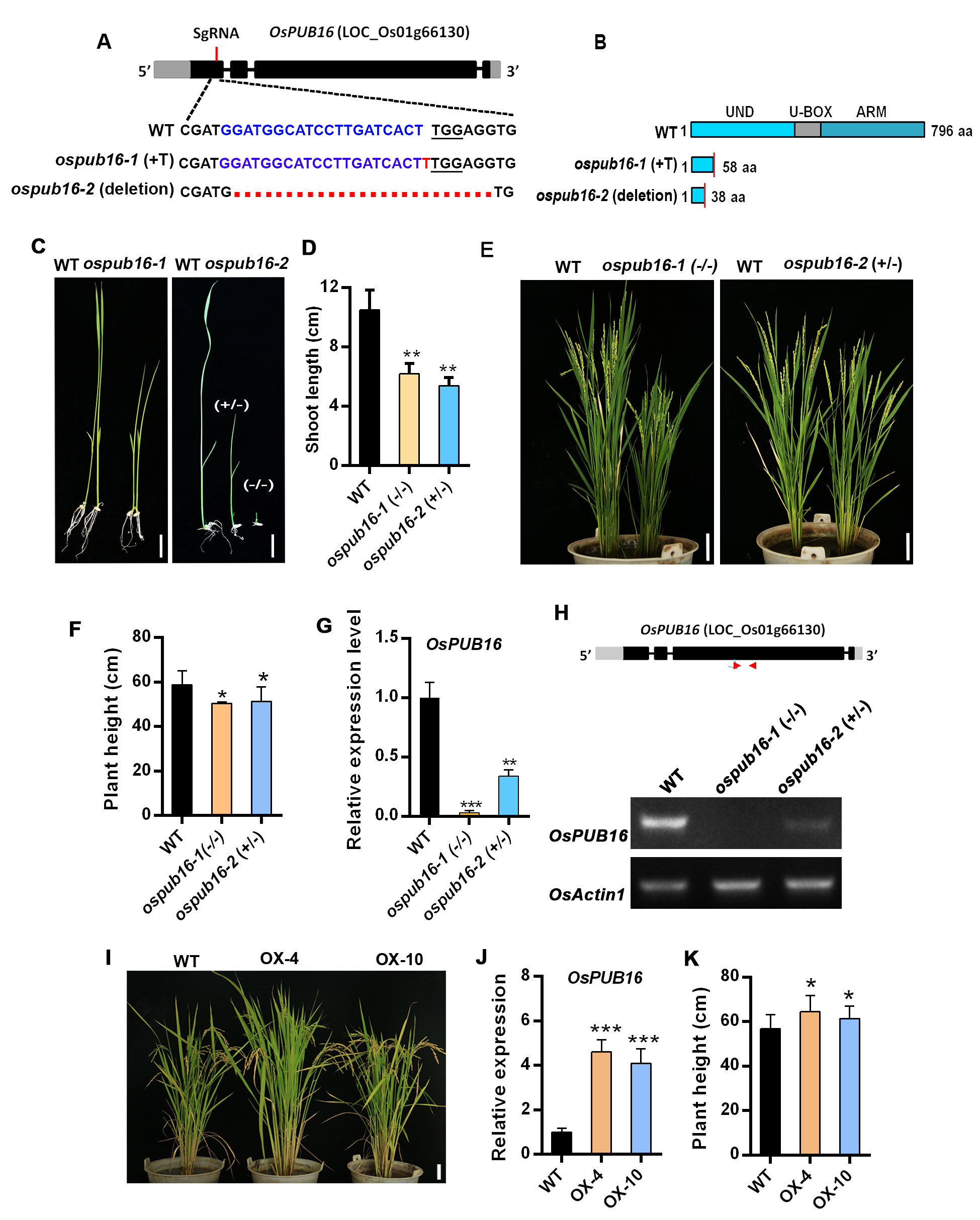

Supplement: S3 Fig — (A) Schematic presentation of the gene structure of OsPUB16 and CRISPR-cas9 editing site. White boxes: untranslated regions; Black boxes: exons; black line: intron. TGG: PAM (protospacer adjacent motif). The CRISPR-cas9 target site and mutation were shown in homozygous mutants of ospub16. The letter in red represents the nucleotide insertion, and the red dots represent nucleotide deletion. (B) Frameshift mutations in ospub16 mutants in (A) resulting in early termination of protein translation. (C) Two-week-old wild type (WT, Nip) and ospub16 mutants. Scale bars, 2 cm. (D) Shoot length measurement for 2-week-old seedlings. Data are means ± SD (n = 10). (E) Three-month-old WT and ospub16 mutants. Scale bars, 10 cm. (F) Plant height measurement for 3-month-old plants. Data are means ± SD (n = 10). (G) Analysis of OsPUB16 by quantitative real-time PCR analysis. The transcript levels in wild type were defined as “1”. Error bars indicate SD (n = 3 biological replicates). OsActin1 was used as the internal control. (H) Analysis of OsPUB16 by semi-quantitative PCR analysis. The red arrows are the positions of primers for quantitative real-time PCR and semi-quantitative PCR analysis. (I) Four-month-old WT and OsPUB16 overexpression (OX-4, OX-10) plants. Scale bar, 10 cm. (J) Expression analysis of OsPUB16 independent OsPUB16 overexpression transgenic lines (OX-4, OX-10). The transcript level in the wild type (Nip) was defined as “1”. Data are means ± SD (n = 3). (K) Plant height measurement for 4-month-old plants. Data are means ± SD (n = 10). In (D), (F), (G), (J) and (K), the significant difference between transgenic plants and wild type was determined by Student’s t test. *p < 0.05, **p < 0.01 or ***p < 0.001. (TIF) [file pgen.1010520.s003.tif]

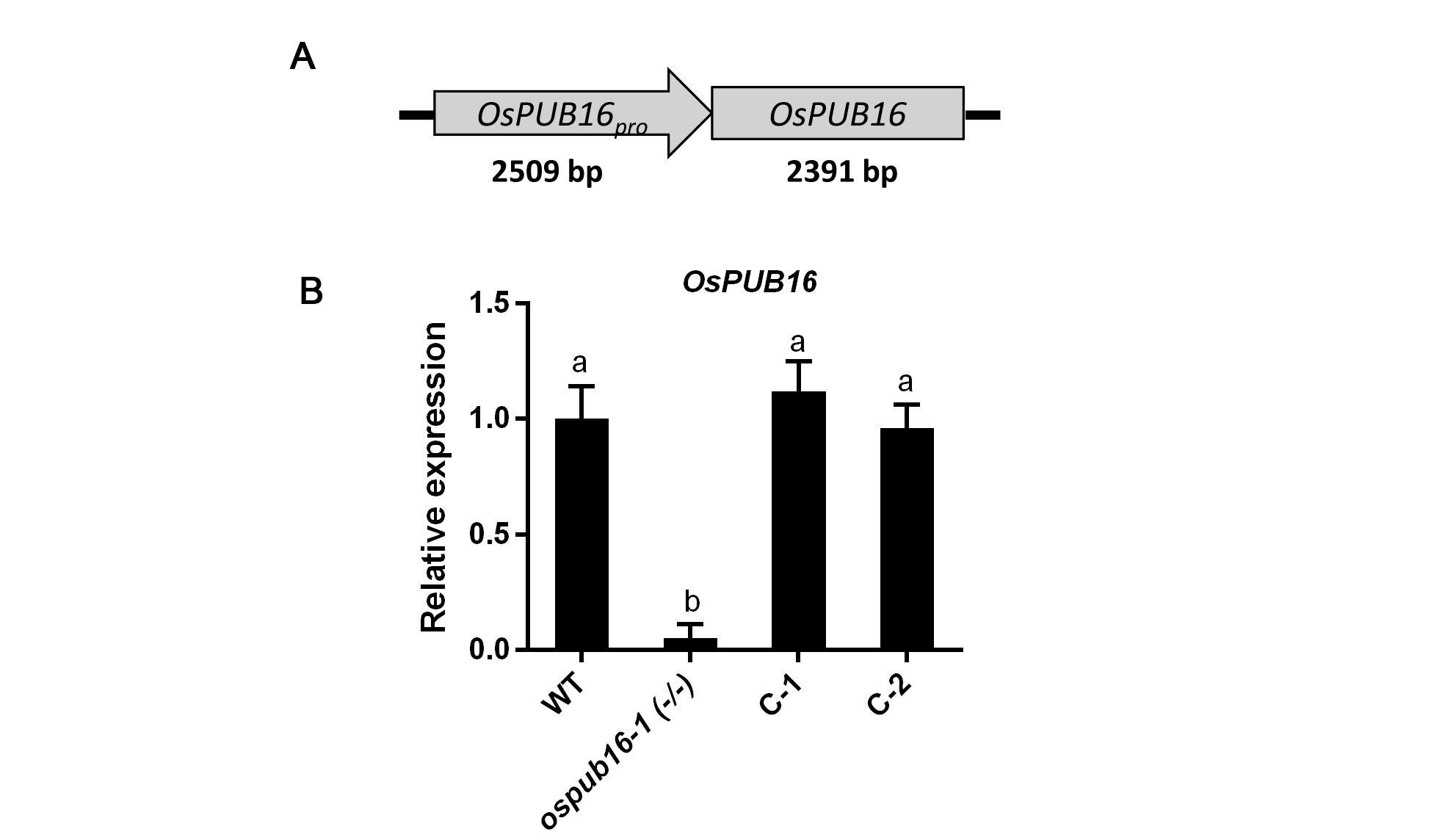

Supplement: S4 Fig — (A) Construction of the complementary vector. The coding sequence of OsPUB16 driven by its own promoter was cloned into pCAMBIA1301. (B) Analysis of OsPUB16 by quantitative real-time PCR analysis in WT, ospub16-1 mutant and complementary lines. The transcript levels in WT were defined as “1”. Error bars indicate SD (n = 3 biological replicates). OsActin1 was used as the internal control. (TIF) [file pgen.1010520.s004.tif]

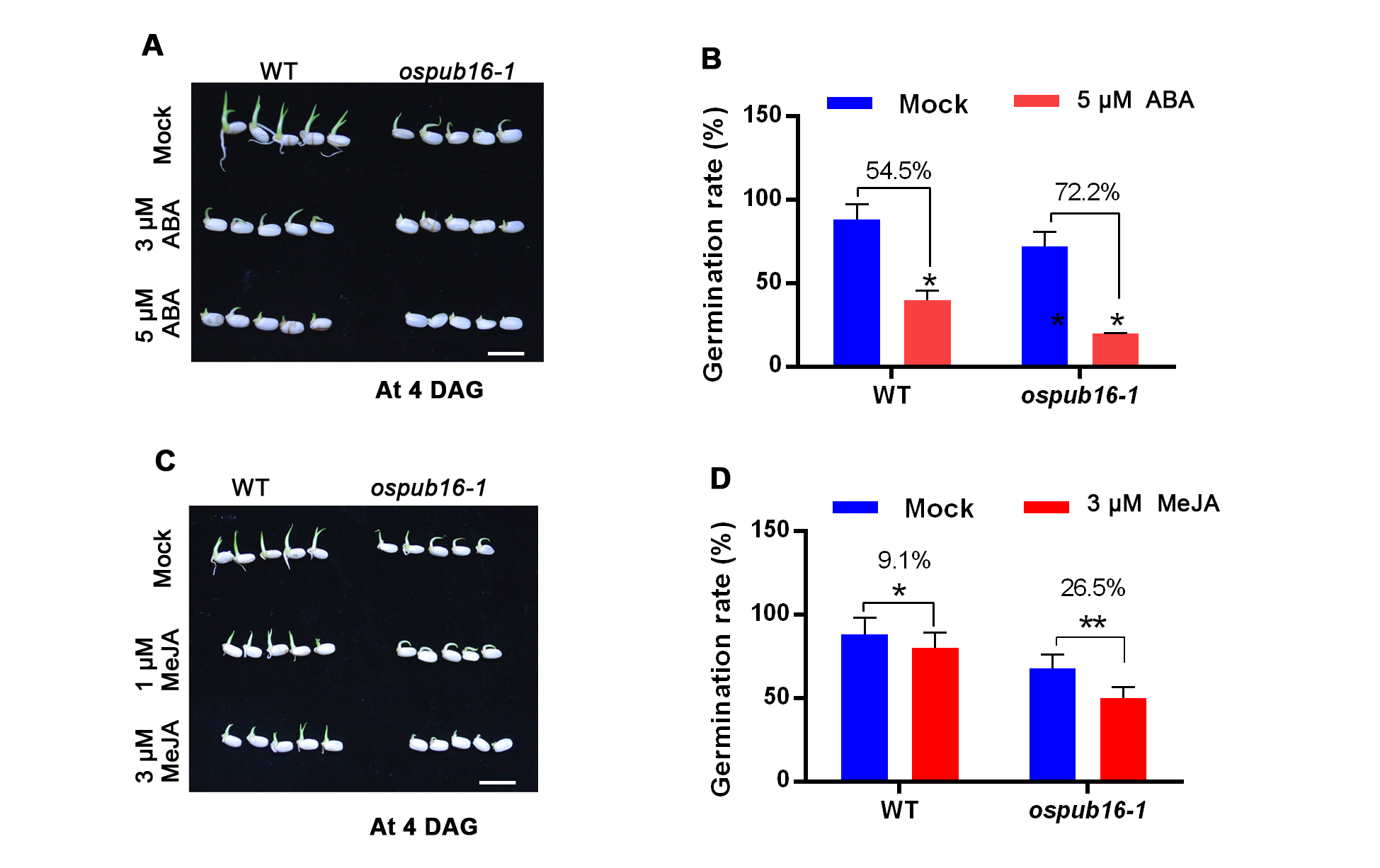

Supplement: S5 Fig — (A, C) Seed germination of WT and ospub16-1 mutant on 1/2MS medium without or with different concentrations of ABA (A) or MeJA (B) at 4 DAG (day after germination). Bars = 1 cm. (B, D) Seed germination rate on 1/2MS medium without or with 5 μM ABA (J) or 3 μM MeJA (L) at 4 DAG. Data are means ± SD with biological triplicates (n = 3, each replicate containing 50 seeds). In (B) and (D), the significant difference between the treated and untreated plants was determined by Student’s t test. *p < 0.05. (TIF) [file pgen.1010520.s005.tif]

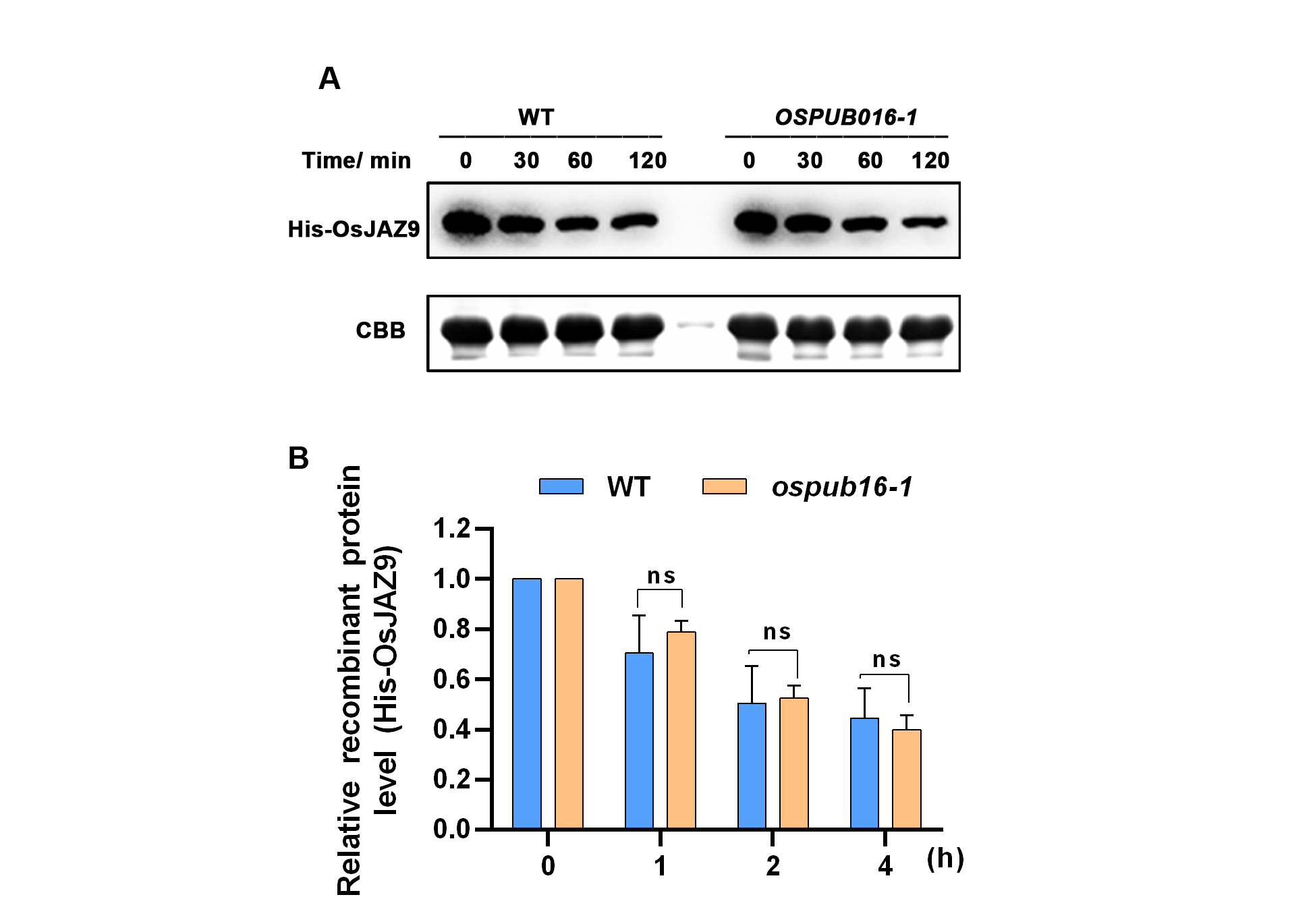

Supplement: S6 Fig — (A) Representative pictures showing the cell-free degradation assay for recombinant His-OsJAZ9, in the presence of 10 mM ATP. Recombinant His-OsJAZ9 was detected by anti-His antibody. The Coomassie blue–stained ribulose-1,5-bisphosphate carboxylase/oxygenase (Rubisco) large subunit (Rbc L) was used as a loading control. (B) Relative recombinant protein levels of His-OsJAZ9 at indicated time points in the cell-free degradation. Data are means ± SD (n = 3, three independent replicate experiments). The significant difference between the treated and untreated was determined by Student’s t test. ns, not significant. (TIF) [file pgen.1010520.s006.tif]

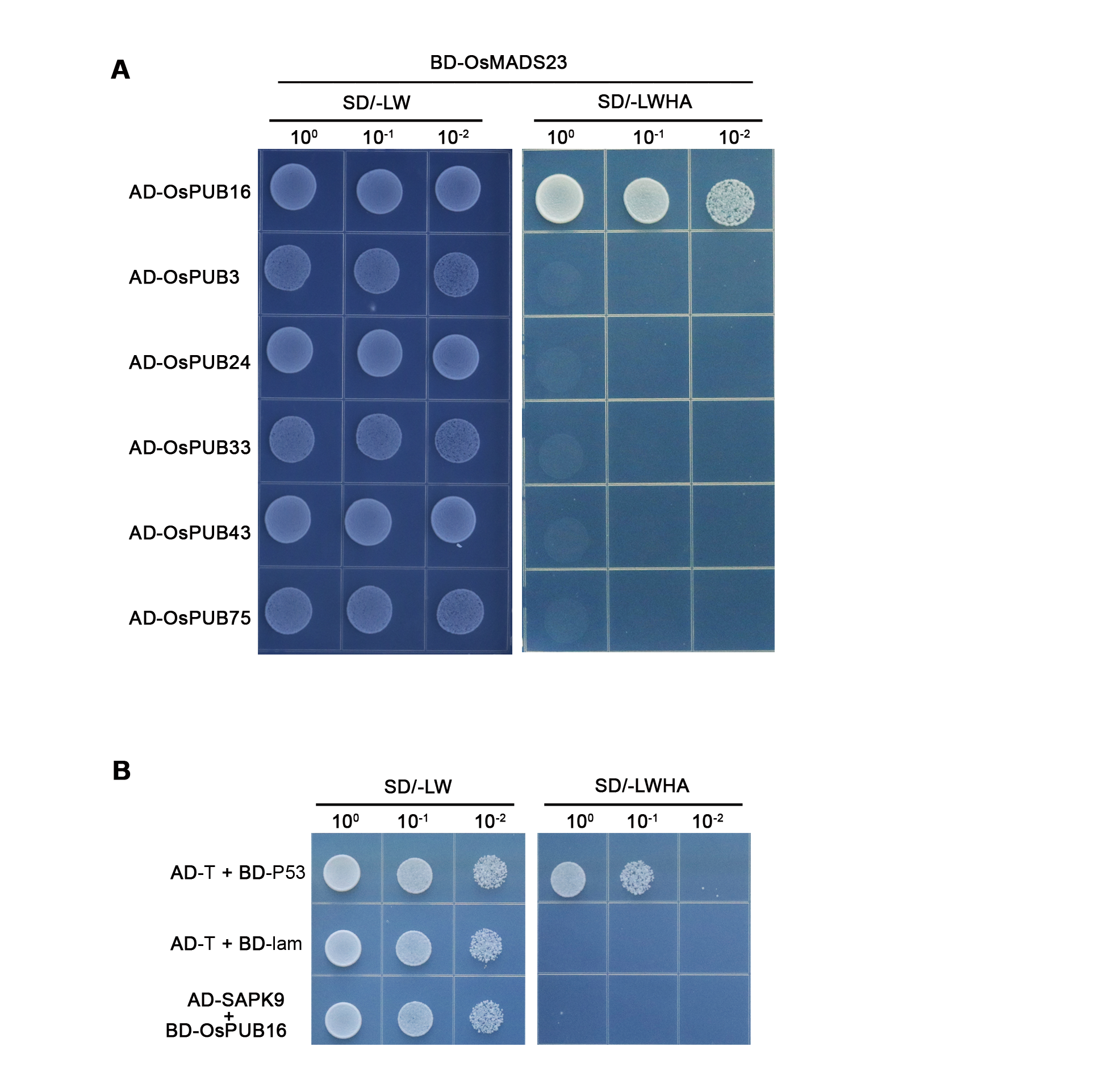

Supplement: S7 Fig — Yeast two-hybrid assays to test the interaction of OsPUB16 with OsMADS23 (A) or SAPK9 (B). Yeast cells were grown on synthetic defined (SD)/-Leu-Trp (-LW) medium and SD/-Ade-His-Leu-Trp (-LWHA) medium. The AD-T + BD-P53 was used as a positive control, and AD-T + BD-lam as a negative control. (TIF) [file pgen.1010520.s007.tif]

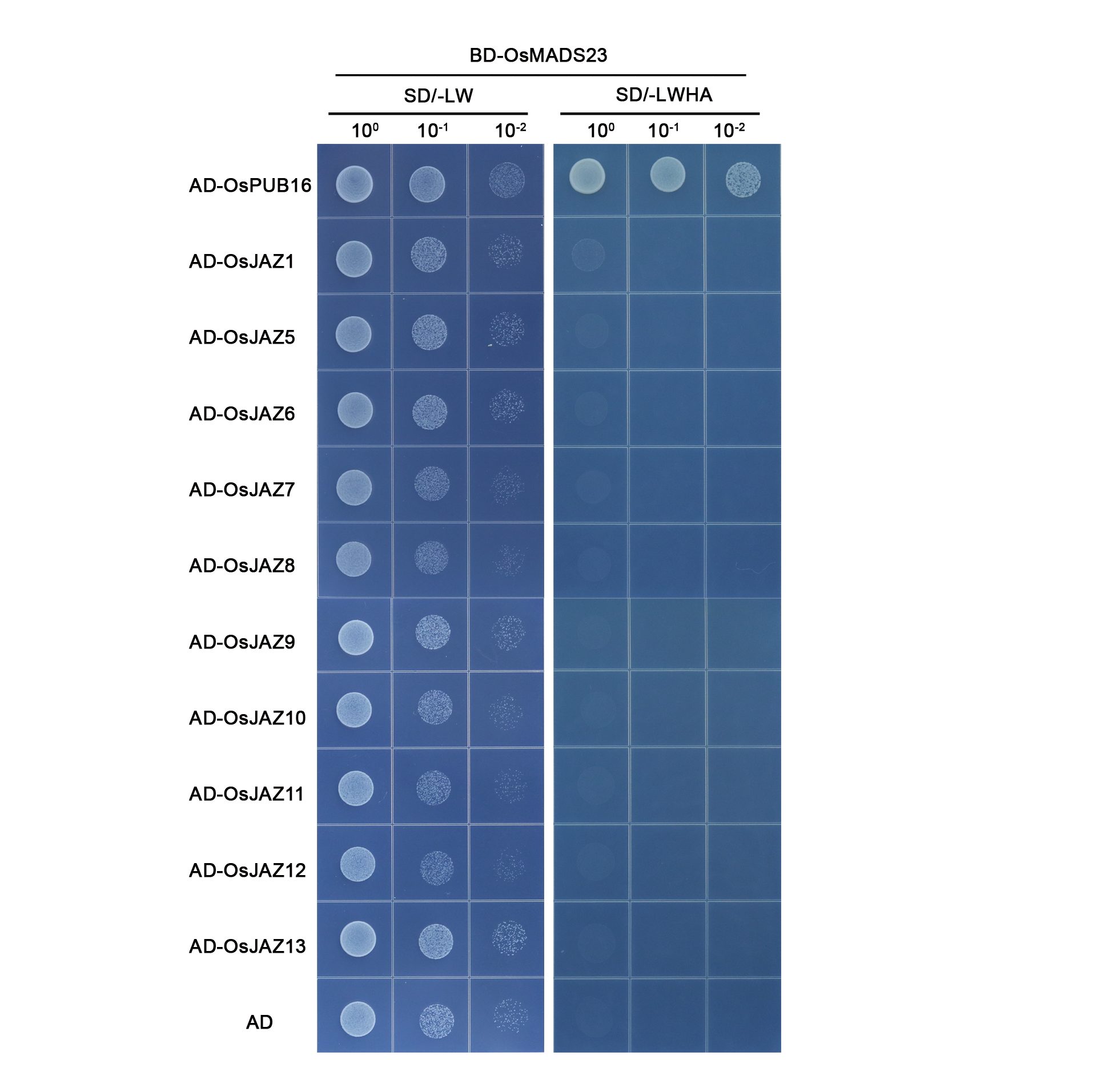

Supplement: S8 Fig — Yeast cells were grown on synthetic defined (SD)/-Leu-Trp (-LW) medium and SD/-Ade-His-Leu-Trp (-LWHA) medium. (TIF) [file pgen.1010520.s008.tif]

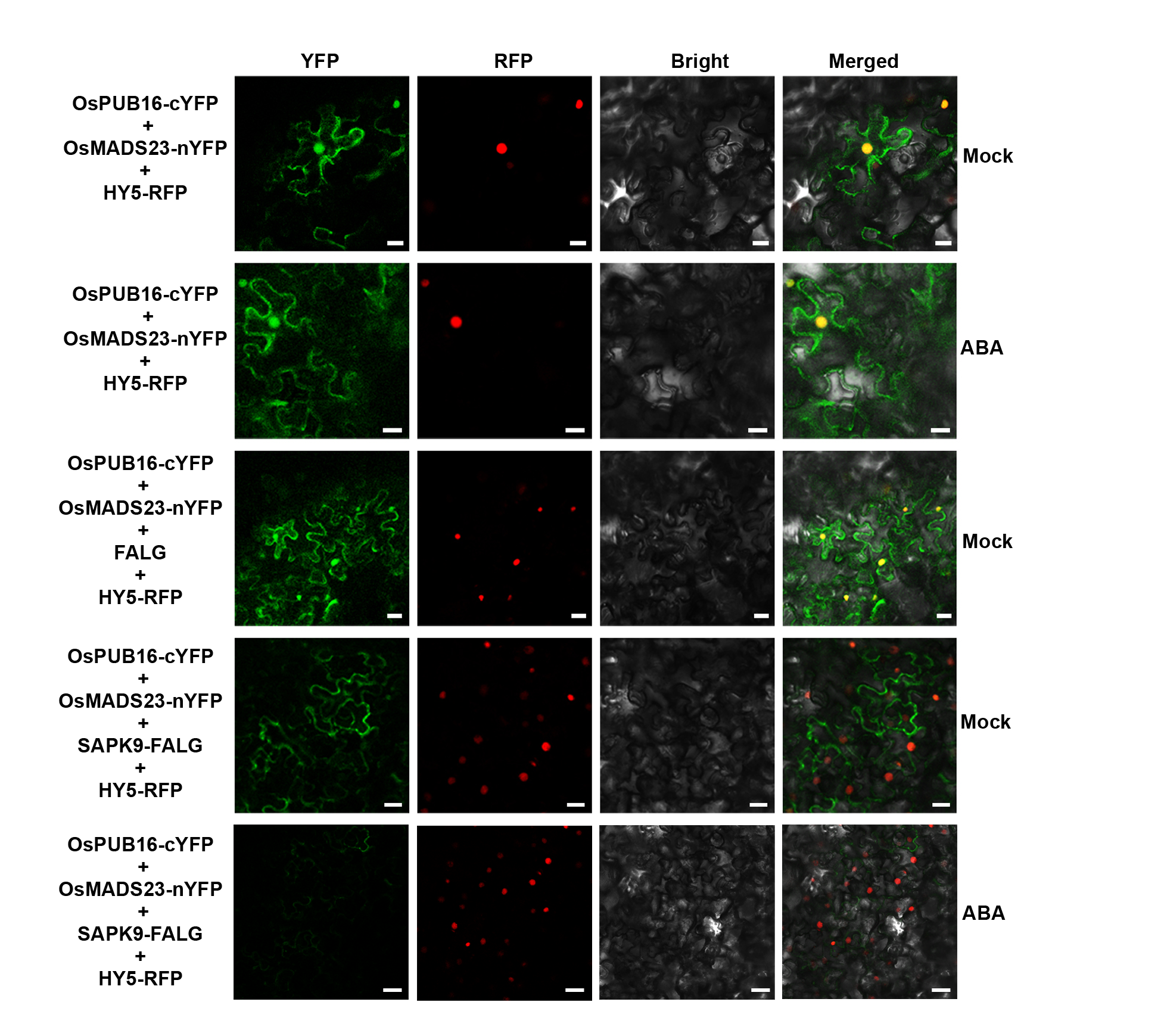

Supplement: S9 Fig — Confocal images showing the interference SAPK9 with the OsPUB16-OsMADS23 interaction, particularly in the presence of ABA. OsPUB16-cYFP and OsMADS23-nYFP were co-expressed in the leaves of Nicotiana benthamiana with or without the presence of SAPK9. Strong YFP fluorescent was detected when OsPUB16-cYFP and OsMADS23-nYFP were co-expressed, but the fluorescent signals were reduced in the presence of SAPK9-FLAG, not FLAG alone. ABA treatment further reduced the YFP fluorescent signals in the leaves co-expressing OsPUB16-cYFP and OsMADS23-nYFP with SAPK9-FLAG, but not in the leaves containing OsPUB16-cYFP and OsMADS23-nYFP. HY5-RFP was used as a nuclear-localized marker. YFP and RFP fluorescence signals were visualized using the confocal microscope (Leica SP8). YFP, fluorescent channel in yellow and RFP fluorescent channel in red. (TIF) [file pgen.1010520.s009.tif]
